# OSBPL3-driven sterol metabolic reprogramming promotes oncogenic signaling and therapeutic resistance in pancreatic cancer

**DOI:** 10.7150/thno.113637

**Published:** 2026-02-18

**Authors:** Qihui Sun, Xiaojia Li, Qi Zou, Yang Chen, Xiaoqi Zhu, Hailin Jiang, Tingting Jiang, Fang Wei, Keping Xie

**Affiliations:** 1 Center for Pancreatic Cancer Research, The South China University of Technology School of Medicine, Guangzhou, Guangdong 510006, China.; 2 Guangzhou Digestive Disease Center, Guangzhou First People's Hospital and The Second Affiliated Hospital, South China University of Technology School of Medicine, Guangzhou, China.; 3 The South China University of Technology Comprehensive Cancer Center, Guangzhou, Guangdong 510006, China.

**Keywords:** Pancreatic cancer, OSBPL3, tumor stemness, therapeutic resistance, immune microenvironment

## Abstract

**Background:**

As a member of the oxysterol-binding protein-like (OSBP) family, which is primarily involved in lipid transport and metabolic regulation, Oxysterol-binding protein-like protein 3 (OSBPL3), has garnered increasing attention due to its abnormal expression and functional roles in various cancers. However, the specific role and molecular mechanisms of OSBPL3 in pancreatic cancer (PDA) remain unclear.

**Methods:**

Single-cell and spatial transcriptomic data analyses combined with functional experiments were utilized to systematically evaluate OSBPL3 expression changes at various stages of PDA. Cell lines with decreased or increased expression of OSBPL3 were generated to analyze its role in cell proliferation, stemness, metastasis and chemoresistance. Single-cell transcriptomic and mass spectrometry data was further integrated with functional validation to explore the regulatory mechanisms through which OSBPL3 modulates PDA malignancy.

**Results:**

OSBPL3 was highly expressed throughout various stages of pancreatic inflammation, precursor lesions, and PDA in both human and mouse pancreatic tissues. Increased OSBPL3 expression significantly enhanced the proliferative capacity and stemness of PDA cells, and promoted their migration, invasion, and metastasis. Moreover, increased OSBPL3 expression impacted on the malignant behaviors of PDA, *e.g.*, reduced PDA cell sensitivity to oxaliplatin, whereas inhibition of NOTCH pathway significantly attenuated the drug resistance and stemness features induced by increased OSBPL3 expression, suggesting that OSBPL3 modulated PDA malignancy via oncogenic pathways such as NOTCH signaling pathway. Furthermore, increased OSBPL3 expression was significantly associated with the enrichment of cholesterol esters and other steroid metabolites, as well as their related pathways. Inhibition of key enzymes involved in cholesterol synthesis resulted in a significant reduction in NOTCH pathway and stemness in PDA *in vivo* mouse models.

**Conclusions:**

Aberrant expression of OSBPL3 plays a pivotal role in PDA initiation and progression and serves as an independent prognostic factor for poor outcomes in PDA patients. OSBPL3 promotes PDA cell proliferation, stemness, and chemoresistance by mediating lipid metabolic reprogramming and regulating oncogenic pathways such as NOTCH. Therefore, inhibition of OSBPL3 expression or blockade of its signaling represent a potential therapeutic strategy to improve therapeutic efficacy and prognosis in PDA patients.

## Introduction

Pancreatic ductal adenocarcinoma (PDA) is one of the most malignant tumors arising from the exocrine pancreatic epithelium and is a leading cause of cancer-related death worldwide 1,2. The intrinsic molecular biology of PDA holds great promise for reflecting the natural course of noninvasive precursor lesions and predicting the progression of PDA 3,4. Despite some progress in the diagnosis and treatment of PDA in recent years, the prognosis remains generally poor due to the disease's subtle early symptoms, rapid progression and limited sensitivity to chemotherapy and radiation therapy 1,5. One of the major barriers to effective chemotherapy is the enrichment of cancer stem cell-like cells (CSCs) within the tumor population 6,7 These CSCs exhibit self-renewal capabilities and are believed to play pivotal roles in therapy resistance, recurrence, and metastasis 8. Notably, CSCs often survive cytotoxic therapies and may be further enriched under chemotherapeutic pressure, activating adaptive survival programs that support tumor regrowth 9. Multiple signaling pathways, including NOTCH, WNT, and Hedgehog, have been implicated in the regulation of CSC phenotypes in PDA, with NOTCH signaling playing a particularly prominent role in sustaining stemness and promoting resistance 6,10. Emerging evidence suggests that CSCs are metabolically distinct from bulk tumor cells, exhibiting marked reprogramming of lipid metabolism, particularly in fatty acid and cholesterol biosynthesis and utilization. Cholesterol metabolism has been shown to regulate CSC properties via direct interactions with key signaling pathways, including NOTCH, WNT, and Hedgehog, which are themselves sensitive to alterations in membrane lipid composition 11,12. For instance, cholesterol-rich membrane domains (lipid rafts) can facilitate the clustering and activation of NOTCH receptors, thereby amplifying downstream transcriptional programs critical for CSC maintenance 13,14. Conversely, dysregulated lipid metabolism can arise as a consequence of aberrant pathway activation, creating a reciprocal feedback loop that further promotes stemness, survival, and chemoresistance. Thus, metabolic reprogramming, especially involving lipid and cholesterol metabolism, has emerged as a hallmark of CSCs and represents a promising therapeutic target for aggressive malignancies such as PDA 15,16.

In this context, oxysterol-binding protein-like protein 3 (OSBPL3)—a member of the OSBP family involved in intracellular lipid sensing and transport—has garnered attention for its oncogenic roles in multiple tumor types 17-19. OSBPL3 is known to regulate cholesterol trafficking and membrane dynamics, and its dysregulation has been associated with tumor progression and drug resistance in certain cancers 20,21. However, its specific role in PDA remains poorly defined 19,22. Given its central involvement in lipid metabolic pathways and potential regulation of signaling cascades such as NOTCH, OSBPL3 may play a crucial role in modulating CSC properties and chemoresistance in PDA. In this study, we aim to elucidate the functional role and regulatory mechanisms of OSBPL3 in PDA progression, with particular focus on its impact on proliferation, migration, invasion, CSC characteristics, and chemoresistance. Through a combination of multi-omics analysis and functional assays, we seek to provide new insights into the metabolic underpinnings of PDA aggressiveness and identify OSBPL3 as a potential prognostic biomarker and therapeutic target.

## Methods and Materials

### Cell lines and culture

The human PDA cell lines PANC-1, CFPAC-1, PK-59 and others were originally imported from the American Type Culture Collection (ATCC, Virgin Islands, USA) and preserved by our laboratory. These cell lines were maintained in DMEM, RPMI-1640 and IMDM medium supplemented with 1% penicillin-streptomycin and 10% fetal bovine serum (FBS) at 37 °C in a 5% CO_2_ incubator, respectively. All of the cell lines were obtained between 2020 and 2022, routinely tested for mycoplasma contamination within the last 6 months by using PCR, and used at passage numbers < 15 for this study after reception or thawing in our laboratory.

### Mouse models

All animal experiments adhered to the guidelines of the Institutional Animal Care and Use Committee of South China University of Technology (#AE-2025082). Adult healthy C57BL/6J mice (6-12 weeks, Hunan SJA Laboratory Animal Co., Ltd) were used for* in vivo* tumor growth assay and the establishment of pancreatic disease models. Two acute experimental pancreatitis (AP) mouse models were established: 1) Caerulein (CAE) -induced acute pancreatitis. AP was induced by intraperitoneal injection of CAE (80 µg/kg, once every h, 8 injections per day, for 2 consecutive days). Control mice were injected with the corresponding volume of saline. The mice were euthanized before injection, and killed 1 and 7 days after the last CAE injection; and 2) pancreatic duct ligation model 23.

To establish orthotopic transplantation model of PDA, 1x10^6^ Panc02 mouse cells were mixed in a 1:1 cell suspension with Matrigel (Corning, New York, USA; Concentration:1:8) and injected into the pancreas of mice. Tumor volumes (formula: length x width^2^/2) were measured weekly, and mice were killed four weeks after tumor cell injection, and tumors were collected for weighing and photography. Stably transfected cells (1x10^6^ cells) in 50 μL medium were subcutaneously injected into left side of the posterior flanks of nude mice. Three days later, oxaliplatin (5 mg/kg) were injected intraperitoneally into mice with one injection every five days. Treatment was continued for a total of 4 cycles and the xenograft tumors were dissected for further experiments.

All mice were maintained in the animal facility at the South China University of Technology under specific pathogen-free (SPF) conditions (#AE-2025082).

### Overexpression plasmid and siRNA transfections

Small interfering RNAs (siRNAs) targeting OSBPL3 (5'-GCAAGAAGAUCUGUGUCAUTT-3') or a negative control (5'-UUCUCCGAAGGUGUCACGUTT-3') were synthesized by GenePharma (Shanghai, China). Overexpression plasmids for both human and mouse OSBPL3 were obtained from GenScript (Hong Kong, China). Transfection of siRNAs or overexpression plasmids was performed using Lipofectamine 3000 (Invitrogen; Thermo Fisher Scientific, Inc. Massachusetts, China) and Opti-MEM Medium (Gibco; Thermo Fisher Scientific, Inc. Massachusetts, China) following the manufacturer's instructions. Transfection efficiency was assessed by qRT-PCR 48 h post-transfection and western blotting 72 h post-transfection.

### qRT-PCR

Total RNA was extracted from cell lines by TRIzol reagent (Omega, Georgia, USA). The cDNA templates converted from RNA were synthesized by a PrimeScript RT Master Mix (Takara, Kyoto, Japan). Next, quantitative real-time PCR (qRT-PCR) using SYBR Green II (Takara, Kyoto, Japan, Country) was conducted with a StepOnePlus RT-qPCR system (Thermo Fisher Scientific, USA). Gene expression was normalized to β-actin, GAPDH,18S, and the relative quantitative values were calculated by the 2-ΔΔCt method 24. mRNA levels were normalized by mRNA levels of β-Actin or GAPDH. The relative quantitative value was expressed as the comparative Ct (2- (ΔCt-Cc)) method. The primers applied in this paper are listed in **Table S1**.

### Western blot analysis

Total proteins were extracted from cultured cells using RIPA lysis buffer (P0013B, Beyotime, Shanghai, China) supplemented with protease inhibitors. After determining the protein concentration, an appropriate volume of SDS sample buffer was added, and the mixture was heated at 100 °C for 15 min. SDS-PAGE gel preparation was performed using an SDS-PAGE Gel Quick Preparation Kit (P0012AC, Beyotime, Shanghai, China). Subsequent steps included sample loading, electrophoresis, transfer to a PVDF membrane, and blocking with 5% non-fat dry milk. Specific antibodies were then applied for incubation. The blots were visualized with the ECL enhanced chemifluorescent reagent (Thermo Scientific, Massachusetts, USA). All primary and secondary antibodies, including their sources and host, are summarized in **Table S2**.

### Data acquisition and processing

The transcriptome profiles (150 pancreatic tumor tissues and 4 matched non-tumor tissues from TCGA dataset, 167 normal pancreatic tissues from GTEx dataset), matching somatic mutation status and clinical data were extracted in the UCSC XENA database (https://xena.ucsc.edu/). To get the validation set, gene expression profiles of bulk RNA sequencing datasets GSE16515, GSE62452, GSE71729 and GSE32672 25-27 were downloaded from the GEO database (https://www.ncbi.nlm.nih.gov/geo/). The comprehensive transcriptome characteristics of PAAD at different stages were investigated in a single cell RNA cohort with GEO code of GSE141017 28. The other scRNA-seq cohorts of 16 PAAD tissue samples and 3 adjacent normal pancreas samples from GSE155698 29, and a spatial transcriptomics of patient primary PDA with GEO ID: GSE203612 30, respectively), were also acquired from the GEO database. Downstream analysis was performed using Seurat V3.2.2 in R Studio V1.3.1093.

### Molecular docking analysis

The molecular docking analysis was conducted to predict the potential interaction between oxaliplatin and OSBPL3. The 3D chemical structure of oxaliplatin was retrieved from the PubChem database (https://pubchem.ncbi.nlm.nih.gov/), and the crystal structure of OSBPL3 (PDB ID: 7CYZ) was obtained from the RCSB Protein Data Bank (https://www.rcsb.org/). Prior to docking, both ligand and protein structures were prepared using Open Babel for format conversion and preprocessing. Water molecules were removed from the protein structure, and polar hydrogen atoms were added. The resulting structures were converted to the PDBQT format, which is compatible with AutoDock Vina. Docking simulations were performed using AutoDock Vina version 1.2.2. The docking grid box was defined to encompass the predicted binding site of OSBPL3, with dimensions set to 40×40×40 Å. The exhaustiveness parameter was set to 8 to ensure a comprehensive search of binding conformations. The docking results were ranked based on binding affinity scores, and the top-ranked conformation was selected for visualization and interpretation using PyMOL.

### Analysis of lipid metabolism and transcriptome data in PDA organoids

We selected the PDA organoid lipid metabolism mass spectrometry dataset (OMIX004117) from the OMIX database (https://ngdc.cncb.ac.cn/omix/) and downloaded the accompanying transcriptome dataset. A total of 20 samples that contained both transcriptome and lipid metabolism mass spectrometry data were extracted for subsequent analysis.

Based on the median expression value of OSBPL3 in the transcriptome data, we divided the 20 samples into high and low expression groups (10 samples each). After loading the mass spectrometry data and grouping data into R, we utilized the “opls” function to perform Partial Least Squares Discriminant Analysis (PLS-DA) and calculated the variable importance in projection (VIP) values. We then computed the fold change (FC) values and p-values to identify differentially expressed metabolites. Additionally, we conducted correlation analysis using the “cor.test” function to determine the metabolites that correlated most strongly with OSBPL3 expression.

To visualize the results, we employed the “Pheatmap” package to generate heatmaps. Furthermore, we utilized the “ggpubr” package's “ggdotchart” function and the “mixOmics” package's “plotLoadings” function for the visualization of selected metabolites.

### Cellular viability and clonogenic assay

Based on the growth rate, PANC-1, PK-59 and CFPAC-1 cell lines with overexpression-OSBPL3 or si-OSBPL3 were selected from the long log-growth stage and seeded on 96-well plates in amounts of 1~5 x10^3^ cells. Then, every 24 h, 10% cell counting kit-8 (CCK-8, Dojindo) solution was added and spectrophotometer was used for the measurement of optical density (OD) value at a wavelength of 450nm.

For colony-formation assay, 5x10^2^ or 1x10^3^ cells were seeded into 6-well plates cultivated in medium supplemented with 10% FBS. The colonies were cleaned with PBS, fixed with 4% paraformaldehyde and stained with crystal violet after 2 weeks. The number of grown colonies was calculated by ImageJ, and images were captured using a microscope (Zeiss, Göttin-gen, Lower Saxony, Germany).

### Transwell assay and invasion assay

The 24-well Transwell chamber was used in transwell migration and invasion assay. After cleaning with PBS, digestion by Trypsin, and resuspension with serum-free medium, cells at a density of 2x10^5^ containing 100 μL of serum-free media were seeded in the upper compartment of the transwell chamber, and 600 µL complete media were filled into the lower chamber. For an invasion assay, the upper transwell chambers were coated with 100 μL mixture of Matrigel and serum-free media at 1:8 dilution. Almost 5x10^5^ cells in serum-free media were seeded on top of the polymerized Matrigel mixture. After incubation at 37 °C for 24 h, when the cells in the upper chamber migrated or invaded the lower compartment under the action of serum gradient, they were fixed were fixed with 4% paraformaldehyde for 15 min and subsequently stained with crystal violet for 30 min at room temperature. Stained cells were thoroughly washed in water and air-dried. Images were taken with an inverted microscope.

### Wound healing assay

To further assess the cell migration, scratch wound assay was applied. 3.5x10^6^ cells were seeded at 6-well plate maintained with complete medium and incubated at 37 °C with 5% CO_2_ to reach well confluence. The monolayer of cells was scratched using a pipette tip to make a cell-free gap. The cells were washed with PBS and cultured in media supplemented with 2% FBS. The photographs of the scratch wound were recorded at 0, 12 and 24 h post-wounding and the closure area of wound was measured using the ImageJ software.

### Tumorsphere formation experiment

Tumorsphere were generated by seeding cells (1~3x10^3^) in 6 well ultra-low attachment plates and cultured in DMEM/F12 (Gibco) supplemented with 100 ng/μL B27 (Invitrogen), 100 ng/μL epithelial growth factor (EGF, Sigma-Aldrich), and 50 ng/μL fibroblast growth factor (FGF, PeproTech) for four days. The size and number of tumorspheres were observed with a microscope and photographed with software. Subsequently, RNA of tumorsphere was extracted for qRT-PCR to verify the expression of stem cells genes.

### Cell cycle analysis

Selected cell lines in the logarithmic growth phase with good viability were treated for 24 h, after which the culture medium was discarded. The cells were washed with PBS, digested with trypsin, and centrifuged before resuspending in 1 mL of PBS. Following cell counting, approximately 1x10^6^ cells were transferred to a 1.5 mL EP tube. The centrifuge was set to 4 °C and 1000 rpm for 5 min. After discarding the supernatant, the cells were resuspended in another 1 mL of PBS and gently pipetted to achieve a single-cell suspension, followed by centrifugation at 300 g for 10 min.

Then, the cells were fixed in chilled 70% ethanol at 4 °C for 12 to 24 h. Staining was performed according to the Biotium cell cycle-apoptosis detection kit instructions. Propidium iodide staining solution was prepared based on the number of samples, and the cells were resuspended and incubated in the dark at room temperature for 30 min. Flow cytometry was conducted within 24 h post-staining to analyze the cell cycle. Subsequently, FSC files for all samples were exported and analyzed using FlowJo software.

### Immunocytochemistry (ICC), Immunohistochemistry (IHC) and Immunofluorescence (IF)

Pancreatic tissue from wild-type, acute pancreatitis, containing CAE, PDL, KC and KPC murine models, and pancreatic cancer models was collected and fixed with formalin, paraffin embedded and sectioned as 4 μm slides for hematoxylin-eosin staining (HE), immunohistochemical and immunofluorescent staining. This staining was performed using the OSBPL3 mouse polyclonal antibody (sc-514097, Santa Cruz Biotechnology, United States) at an antibody dilution of 1:100. Sections were stained with HE according to standard procedures. Slices are roasted at 65 °C for 1-2 h, then transferred to xylene to deparaffinization and gradient alcohol to rehydrate, respectively. Subsequently, some slides were stained with HE, and the corresponding continuous sections were stained with IHC or IF. For HE staining, the sections were immersed in Hematoxylin for 10 sec, stained with EOSIN for ~30 sec, dehydrate in ascending alcohol solutions, and cleared with xylene.

For IHC assay, antigen retrieval with Tris/EDTA pH 9.0 buffer or sodium citrate pH 6.0 was used to expose the antigenic sites in order to allow the antibodies to bind. To suppress endogenous peroxidase activity, the slides were incubated in 3% H_2_O_2_ in TBST for 15 min at room temperature. Then, the slides were blocked in 10% goat serum in TBST for 2 h. After incubation of primary antibody with appropriate concentration overnight, rinsing the slides 3 times with TBST for 5 min, incubating with secondary antibody, developing the solution of 3,3' -diaminobenzidine (DAB), staining with hematoxylin, followed by cleaning, dehydrating and mounting. IF staining assay goes through similar steps of antigen retrieval, blocking and antibody incubation as immunohistochemistry. However, it should be noted that in immunofluorescence, secondary antibody incubation and subsequent steps need to be performed away from light, and DAPI is required for sealing.

For ICC staining assay, cultured cells with or without treatment were fixed using 4% paraformaldehyde for 15 min. After permeabilizing the cells in PBS supplemented with 0.1% Triton® X-100 and blocking with PBS with 1% BSA. After sample preparation, the antibody incubation and counterstaining and mounting were also done. All slides were automatically captured from 420 to 720 nm at 20-nm intervals with the same exposure time.

### Cellular triglyceride and total cholesterol quantification

After the indicated treatments, culture media were removed and cells were rinsed three times with phosphate-buffered saline (PBS). CFPAC-1, PK-59, and PANC-1 cells were then harvested by trypsinization and collected by centrifugation. Intracellular total cholesterol (TC) and triglyceride (TG) concentrations were determined using a Total Cholesterol (TC) Content Assay Kit (Solarbio, Cat. No. BC1980, Beijing, China) according to the manufacturer's protocol. Protein concentrations were normalized using a BCA Protein Assay Kit, and the final TG and TC levels were expressed relative to total protein content. Each experiment was independently repeated three times to ensure reproducibility.

### Analysis on the response of drug therapy

The Oxaliplatin (Compound CID: 9887053) was purchased from MedChemExpress (Bellingham, WA, USA) and then diluted to 5 mM in DMSO. Data on cell line gene expression and drug sensitivity. We used version 17a of the Genomics of Drug Sensitivity in Cancer (GDSC) data, comprised of molecular data for 1001 cell lines and 265 anticancer drugs, specifically microarray gene expression data (ArrayExpress accession: E-MTAB-3610) and the IC 50 values for each drug-cell line combination. For computing pan-cancer associations, we used the subset with TCGA-like cancer type label, leaving 805 cell lines. We downloaded the Cancer Cell Line Encyclopedia (CCLE) microarray gene expression and drug sensitivity data from the CCLE web page (https://portals. broadinstitute.org/ccle). For TCGA-PAAD data, we used drug profiling data version and drug metadata version.

### Differential gene expression analysis

Differential expression analysis of the TCGA-PAAD transcriptome sequencing dataset was primarily conducted using the “edgeR”, “DESeq2”, and the “voom” function from the “limma” package, all based on the Negative Binomial Generalized Linear Model (GLM). The differentially expressed genes identified by these three methods were used for subsequent pathway enrichment analysis. In single-cell datasets, differential gene analysis was performed mainly using the “FindMarkers” and “FindAllMarkers” functions from the Seurat package.

### Pathway enrichment analysis

The log-transformed gene expression matrix was prepared as the input file, and the “gsva” function was utilized to obtain GSVA scores for each sample. Visualization of the gene set scoring matrix was conducted using the “Pheatmap” package or other standard methods.

The irGSEA method integrated gene set scoring for single-cell datasets using various algorithms, including AUCell, UCell, singscore, and ssGSEA, based on ranks 31. After loading the “UCell” and “irGSEA” R packages, gene set scoring was performed using the “irGSEA.score” function. The “irGSEA.integrate” function was then used to obtain differentially expressed gene sets. Visualization was achieved using functions such as “irGSEA.heatmap.plot,” “irGSEA.bubble.plot,” “irGSEA.barplot.plot,” and “irGSEA.density.scatterplot.”

### CytoTRACE analysis

Processed single-cell expression count data was utilized to determine the number of detectable genes in each cell. Following the re-normalization of the gene expression matrix, it was converted into relative transcript counts. Pearson correlation analysis was performed between gene expression and gene counts, leading to the identification of the top 200 genes positively correlated with gene counts. The average expression values of these genes were defined as Gene Count Features (GCS). The normalized expression matrix was subsequently transformed into a Markov matrix to assess local similarities among cells. The Non-Negative Least Squares (NNLS) algorithm was then applied to the GCS, with iterative adjustments yielding a predicted order of cellular differentiation. Finally, the “plotCytoTRACE” function was employed to visualize the predicted differentiation sequence alongside the CytoTRACE-associated genes 32.

### Pseudotime trajectory analysis

To fully clarify the gene expression pattern and evolutionary trajectory at each stage of PDA progression, we applied Slingshot algorithm to construct the cell differentiation lineage structure and infer the pseudo-time changes of different lineage cells. After pre-processing, we convert the Seurat object to the SingleCellExperiment object based on the R “SingleCellExperiment” package. Then, the coordinate matrix of the cell and the label vector of the cell cluster are constructed as input files, and the differentiation trajectories were calculated and constructed through the “slingshot” package 33. UMAP was again used for dimensionality reduction.

### InferCNV analysis

After loading the single cell data set of tumor progression in KC mouse model processed by Seurat standard procedure, R package “GetAssayData” was used to extract the expression matrix file. Then AnnoProbe package was applied to prepare gene chromosome annotation file containing gene name, chromosome name, gene start position and gene end position. After the above three input files are obtained, the CTRL sample cells in the data set are used as the reference normal cell population, 17D (17 days), 6W (6 weeks), 3M (3 months), 5M (5 months), 9M (9 months) and 15M (15 months). The sample cells of 6 stages were used as target cell groups, and the inferCNV object was created using the “CreateInfercnvObject” function in the “inferCNV” package. After that, run inferCNV through inferCNV: run. This process mainly goes through the steps of filtering low-expressed genes according to cutoff values, data standardization, log conversion, subtracting reference cell expression values, extreme value processing, chromosome smoothing processing, decentralization, subtracting CNV mean of reference data, logarithmic exponential conversion and noise reduction, so as to reduce the residual signal of normal cells and amplify the signal in tumor cells. After the above processing, we obtain the final gene CNV matrix and related heat maps, and all the results are stored in the specified output folder. The “plot_cnv” function of “inferCNV” package was applied to display the relative expression intensity of each cell in each chromosome region through heat maps.

### Cholesterol modulation and NOTCH signaling assay

To investigate the relationship between OSBPL3-mediated cholesterol metabolism and NOTCH pathway activation, we conducted cholesterol modulation experiments using a cholesterol synthesis inhibitor and an oxysterol supplement. Simvastatin (MK 733; https://www.medchemexpress.cn/Simvastatin.html), a widely used HMG-CoA reductase inhibitor, was employed to block endogenous cholesterol biosynthesis. OSBPL3-overexpressing PK-59 and PANC-1 cells were treated with 15 nM simvastatin for 24 hours. Conversely, to restore intracellular cholesterol levels, 25-hydroxycholesterol (25-OHC; https://www.medchemexpress.cn/25-Hydroxycholesterol.html) was used to treat OSBPL3-knockdown PK-59 and CFPAC-1 cells at a final concentration of 2.5 μM for 24 hours.

Following treatment, cells were harvested, RNAs were used in qRTPCR, and protein lysates were subjected to Western blot analysis. The expression levels of key NOTCH signaling markers, including the NOTCH intracellular domain (NICD) and HES1, were assessed using specific primers and antibodies.

### Data statistics and visualization

All statistical analyses were conducted using R (version 4.1.2) and GraphPad PRISM software (version 9.3.0). Differential expression analysis between cell groups was performed using a two-sided Wilcoxon rank-sum test, followed by Bonferroni FDR correction. Graphical representations were created using R package software. The Wilcoxon test was applied to assess statistical differences between groups. The log-rank test was used to evaluate the statistical significance of survival differences. A p-value of < 0.05 was considered statistically significant.

## Results

### OSBPL3 is highly expressed in PDA and associated with a poor prognosis in patients with PDA

**Figure 1:**
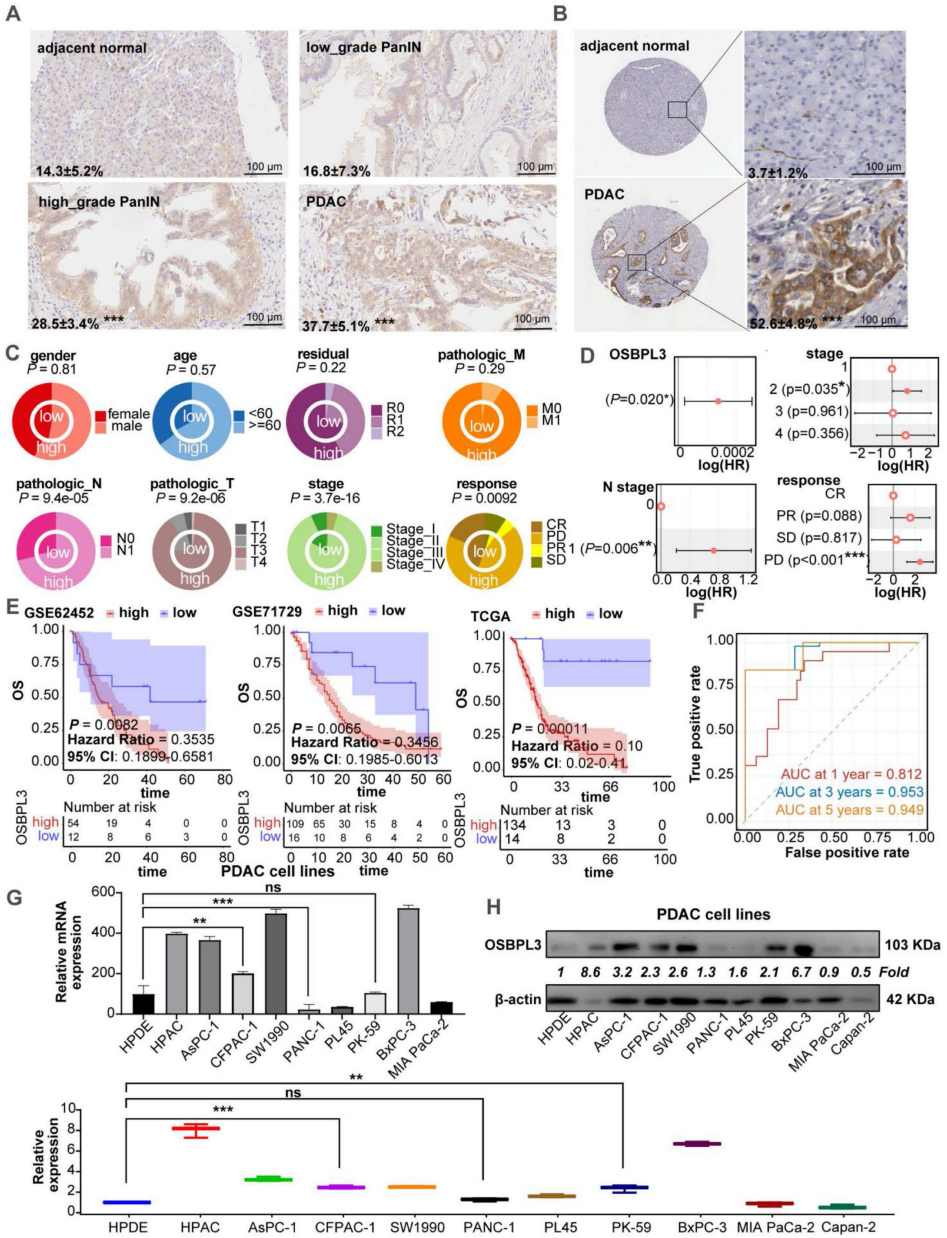
** OSBPL3 is overexpressed in PDA and correlates with poor prognosis.** (**A**) IHC staining of OSBPL3 in human PDA tissue samples (n = 3), showing expression in acinar cells, low-grade and high-grade PanIN, and tumor cells. The data represent three independent experiments; *P < 0.05, **P < 0.01, ***P < 0.001, ****P < 0.0001. (**B**) IHC analysis of OSBPL3 expression in normal pancreas and PDA tissues from the Human Protein Atlas (HPA) database (n = 3). The data represent three independent experiments; *P < 0.05, **P < 0.01, ***P < 0.001, ****P < 0.0001. (**C**) Concentric circle plot illustrating the clinical data distribution between patients with low and high OSBPL3 expression (TCGA-PAAD cohort; n = 148). The inner circle represents the low-expression group, while the outer circle denotes the high-expression group. (**D**) Univariate Cox regression analysis of OSBPL3 expression and clinical factors in PDA (TCGA-PAAD cohort; n = 148). (**E**) Kaplan-Meier survival curves for patients with high or low OSBPL3 expression levels from the GSE62452 (n = 66), GSE71729 (n = 125), and TCGA-PAAD (n = 148) datasets. (**F**) Time-dependent receiver operating characteristic (ROC) curve analysis at 1, 3 and 5 years, illustrating the area under the curve (AUC) values for a multivariate model incorporating OSBPL3 expression and other independent prognostic factors (TCGA-PAAD cohort; n = 148). (**G**) qRT-PCR and (**H**) Western blot analyses of OSBPL3 expression in a panel of PDA cell lines and a normal pancreatic acinar cell line (hTERT-HPDE); The quantification of Western blot results from three independent experiments is presented in the accompanying bar graphs; *P < 0.05, **P < 0.01, ***P < 0.001, ****P < 0.0001.

We performed IHC analysis on human PDA tissues, revealing higher OSBPL3 expression in PanIN and PDA tissues compared to adjacent normal acinar cells (**Figure 1A**). Analysis of PDA tissue from the HPA database showed significantly elevated OSBPL3 expression in tumor regions relative to matched normal tissues (**Figure 1B**). After standardizing and processing the GSE155698 single-cell transcriptome dataset, which includes PDA and adjacent tissue samples, we identified 26 distinct cell clusters, annotated as T cells, acinar cells, and 15 other cell types (**Figure S1A; Table S3**) 29. OSBPL3 expression was notably higher in metaplastic and tumor cells within tumor tissues than that in para-cancerous tissues. We further analyzed OSBPL3 expression using the GSE226829 spatial transcriptomic dataset, which includes data from both normal pancreas and PDA tissues. OSBPL3 expression was significantly higher in the PanIN and tumor cell regions than that in acinar cell regions (**Figure S1B**), a finding corroborated by protein expression data from the CPTAC dataset (*P* = 3.4e-50) (**Figure S1C**). Also, the results from four GEO transcriptome-chip datasets showed that high expression of OSBPL3 in PDA tissues (**Figure S1D**).

We then focused our study on the clinical implications of OSBPL3. Increased OSBPL3 expression was positively associated with advanced clinical stages and poorer treatment responses (**Figure 1C**). The proportion of patients with high OSBPL3 expression at a high stage was significantly higher (*P*=3.7e-16). Patients with high OSBPL3 expression also had relatively higher N1 stage and T3/T4 stage (*P*=9.4e-05, *P*=9.2e-06). For treatment response, there was also less complete response (CR) and more stable disease (SD) and progressive disease (PD) in the group with high OSBPL3 expression in contrast with the low OSBPL3 expression (*P*=0.0092). The results of the COX regression analysis indicated that OSBPL3 and tumor stage, among other factors, were risk predictors for PDA (**Figure 1D**). In addition, patients with high OSBPL3 expression in the three datasets all had shorter overall survival (OS) (**Figure 1E**). The risk factor density plot in **Figure S1E** also shows the same result. Multivariate COX regression model was established for these factors, and ROC curve analysis was performed. The one-year AUC value was 0.823, the three-year AUC value was 0.953, and the five-year AUC value was 0.949 (**Figure 1F**). Nomogram also further confirmed the prognostic efficacy of OSBPL3 in PDA (**Figure S1F**).

We also assessed OSBPL3 mRNA and protein levels in various PDA and normal acinar cell lines using qRT-PCR and western blot. As shown in **Figure 1G**-**H**, OSBPL3 was highly expressed in pancreatic tumor cells such as HPAC, AsPC-1, CFPAC-1, SW1990, BxPC-3 and others compared to the normal pancreatic ductal epithelial cell line hTERT-HPDE.

### Elevated expression of OSBPL3 is significantly enriched in pancreatitis and PDA in mice

**Figure 2:**
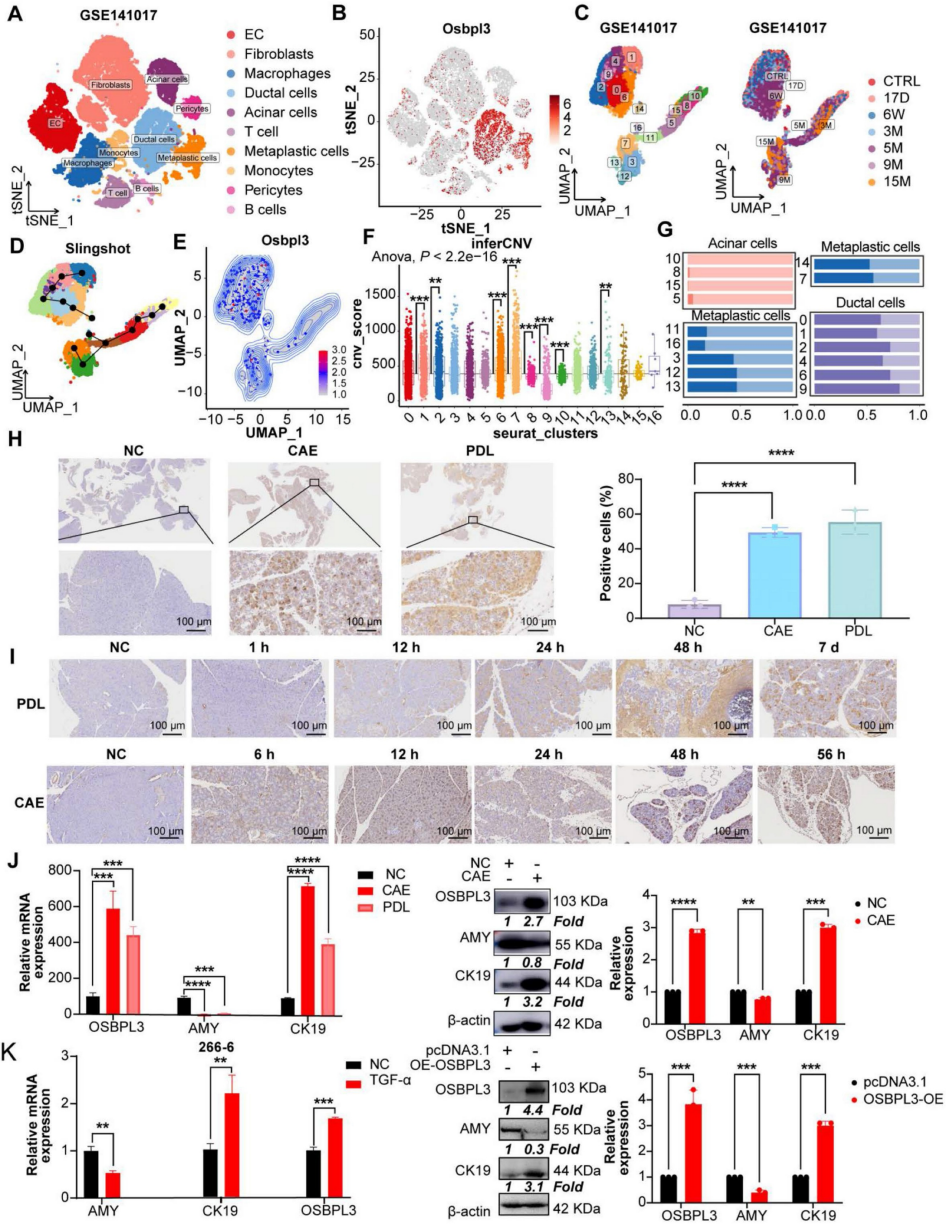
** OSBPL3 is significantly enriched in mouse models of pancreatitis and PDA.** (**A**) t-SNE plots showing cell clustering at seven time points before and after tumor formation in KC mice from GSE141017 dataset. (**B**) The expression of OSBPL3 in various cell subpopulations (cohort: GSE141017). (**C**) UMAP plot of 17 epithelial subpopulations identified through clustering and dimensionality reduction in the GSE141017 dataset, with sample source information indicated. (**D**) Pseudotime trajectory analysis of epithelial cells using the Slingshot algorithm, illustrating the differentiation path. (**E**) UMAP density plot illustrating the expression of Osbpl3 in pancreatic epithelial cells. (**F**) Box plot illustrating inferCNV-derived malignancy scores across different epithelial subpopulations. Statistical significance was assessed using the Anova test, with the overall group mean as a reference; *P < 0.05, **P < 0.01, ***P < 0.001, ****P < 0.0001. (**G**) Proportion of cells with high or low Osbpl3 expression in different epithelial subpopulations after pseudotime ordering. (**H**) Representative IHC staining images of OSBPL3 expression in CAE and PDL mouse acute pancreatitis models. The percentage of positive cells was quantified and visualized using bar plots. n = 3 biological replicates. Data are presented as mean ± SD. (**I**) Representative images of OSBPL3 expression at different time points during the CAE and PDL model development. (**J**) qRT-PCR and Western Blot analyses showing the relative expression of OSBPL3, AMY, and CK19 to β-actin in two acute pancreatitis mouse models (CAE and PDL) compared to normal control samples. (**K**) qRT-PCR analysis shows the expression of OSBPL3, AMY, and CK19 relative to β-actin after TGF-α-induced acinar-to-ductal metaplasia in the 266-6 cell line. Western Blot analysis displaying the relative expression of OSBPL3, AMY, and CK19 to β-actin before and after OSBPL3 overexpression in the pancreatic acinar cell line 266-6. Bar plots showing quantification of Western blot results from three independent experiments. Data are presented as mean ± SD. Statistical significance was determined by Student's t-test, *n* = 3 biological replicates.

To explore the expression dynamics of OSBPL3 during disease progression, we analyzed the GSE141017 dataset, which includes pancreatic samples from *Ptf1a-CreER*, *LSL-Kras^G12D^*, *LSL-tdTomato* (PRT) mice at six time points spanning from the pre-invasive to cancer stages (**Figure 2A**) 28. The marker genes for 10 cell clusters are shown in **Figure S2A**-**B**. Subpopulation analysis revealed significantly higher OSBPL3 expression in metaplastic and ductal cells than that in acinar cells, consistent with the observations in human single-cell datasets (**Figure 2B**-**C**). These findings were further corroborated in two GEO transcriptomic datasets of mouse pancreatitis and PDA (**Figure S2D**).

Next, we categorized epithelial cells into 17 clusters (**Figure 2C** and **Figure S2E**) and used Slingshot trajectory analysis to define the transcriptional states and trace the transition from non-malignant to malignant epithelial cells. The trajectory revealed a progression from cluster 10 to cluster 7 (**Figure 2D**). Malignant cells, identified using the inferCNV algorithm, showed an increase in malignancy corresponding to higher OSBPL3 expression (**Figure 2E-F**, **Figure S2F**). Based on OSBPL3 expression levels, cells were divided into high and low expression groups. Most acinar cells (clusters 10, 8, 15, and 5) exhibited low OSBPL3 expression (**Figure 2G**). In contrast, metaplastic cell subsets showed a gradual increase in the proportion of cells with high OSBPL3 expression as malignancy progressed. In subgroup 7, the proportion of cells with high OSBPL3 expression exceeded those with low expression. IHC analysis of the PDL and CAE models confirmed a time-dependent increase in OSBPL3 expression, correlating with disease progression (**Figure 2H**-**I**).

After identifying significantly elevated OSBPL3 expression in metaplastic and tumor cells from human datasets, we validated these findings using two *in vivo* acute pancreatitis models (CAE and PDL) and an* in vitro* TGF-induced 266-6 cell line model. Both pancreatitis models showed reduced AMY (an acinar cell marker) expression, elevated CK19 (a ductal cell marker) expression, and increased OSBPL3 levels, with β-actin as the internal control (**Figure 2J**). In the 266-6 cell line, OSBPL3 overexpression led to decreased AMY and increased CK19 and OSBPL3 expression (**Figure 2K**). Similarly, TGF-induced acinar-to-ductal metaplasia (ADM) in the 266-6 cell line resulted in reduced AMY and increased CK19 and OSBPL3 levels. These findings highlight the critical role of OSBPL3 in the progression and malignancy of pancreatic disease.

### OSBPL3 promotes proliferation of PDA Cells

**Figure 3:**
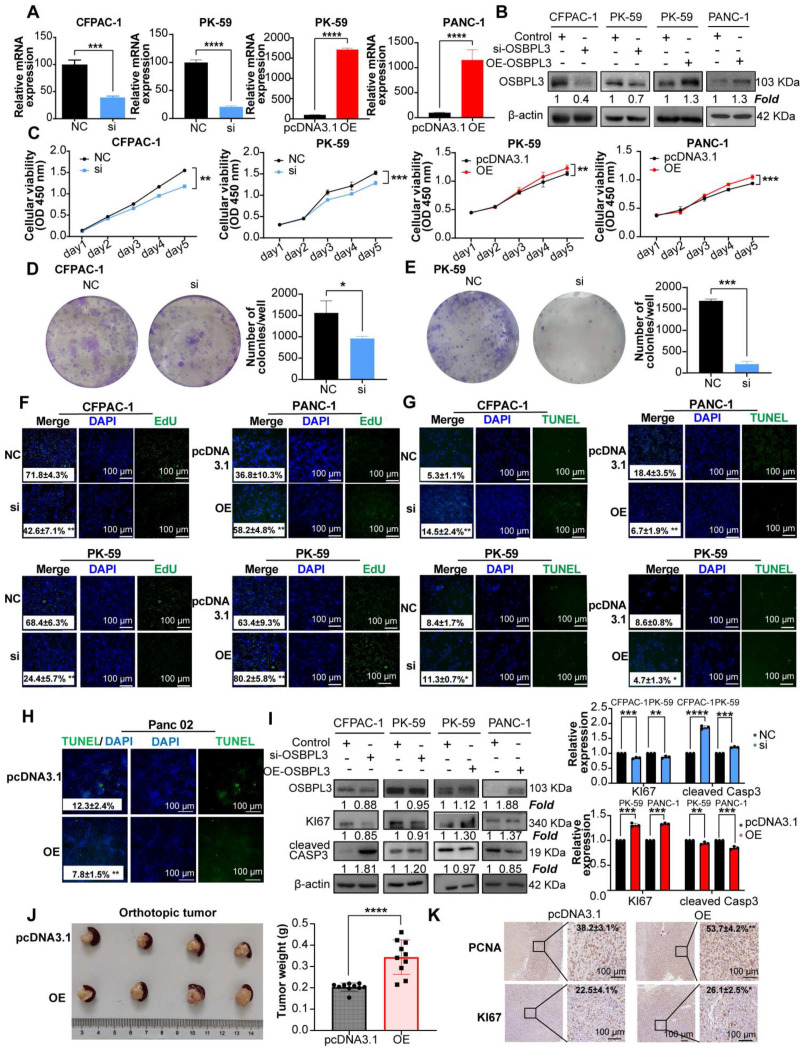
** Aberrant overexpression of OSBPL3 promotes PDA cell proliferation.** (**A**) qRT-PCR validation results of OSBPL3 knockdown using si1 in CFPAC-1 and PK-59 cell lines, and OSBPL3 overexpression in PK-59 and PANC-1 cell lines (n = 3). (**B**) Western blot analysis verifying the knockdown of OSBPL3 using si1 in CFPAC-1 and PK-59 cell lines, and the overexpression of OSBPL3 via plasmid transfection in PK-59 and PANC-1 cell lines. (**C**) CCK-8 assay assessing cell proliferation for 5 consecutive days *in vitro*, comparing OSBPL3 knockdown (Si) and control (NC) in CFPAC-1 and PK-59 cells, and OSBPL3 overexpression (OE) and control (P3.1) in PANC-1 and PK-59 cells. (**D**) and (**E**) Representative images and statistical analysis of colony formation assays in cells with OSBPL3 knockdown or overexpression, showing colony formation before and after transfection (n = 3). The data represent three independent experiments. *P < 0.05, **P < 0.01, ***P < 0.001, ****P < 0.0001. (**F**) EdU proliferation assay to evaluate cell proliferation activity before and after OSBPL3 knockdown or overexpression. DAPI (blue) marks the cell nuclei, while green EdU fluorescence highlights cells in the S phase. Merge represents the combined image from both channels. Representative images of OSBPL3 knockdown or overexpression are shown. Magnification: 20x. (**G**) and (**H**) TUNEL apoptosis assay to assess cell apoptosis before and after OSBPL3 knockdown or overexpression in tumor cell lines. DAPI (blue) marks the cell nuclei, and green TUNEL fluorescence indicates DNA fragmentation during apoptosis. Merge represents the combined image from both channels. Representative images of OSBPL3 knockdown or overexpression are shown. Magnification: 20x. (**I**) Western blot analysis of cell proliferation and apoptosis marker proteins in cells with OSBPL3 knockdown or overexpression. Statistical significance was determined by Student's t-test. *P < 0.05, **P < 0.01, ***P < 0.001, ****P < 0.0001. (**J**) Tumorigenesis in a mouse pancreatic orthotopic tumor model after transfection of Osbpl3 overexpression plasmid into Panc02 PDA cells (two groups; n = 10). Representative images of tumor formation and statistical analysis. Non-paired Student's t-test, *P < 0.05, **P < 0.01, ***P < 0.001, ****P < 0.0001. (**K**) Representative images of IHC staining of the cell proliferation markers MKI67 and PCNA.

After the successful construction of PDA cell lines with knockdown and overexpression of OSBPL3, we subsequently explored the role of OSBPL3 in the malignant biological behaviors of PDA (**Figure 3A**-**B**). Cell viability assays (CCK8) and colony formation experiments demonstrated a significant decrease in cell viability and colony formation in PDA cell lines following OSBPL3 knockdown, while overexpression of OSBPL3 resulted in the opposite effect (**Figure 3C-E**). Additionally, EdU and TUNEL assays were used to revalidate the pro-proliferative role of OSBPL3 in PDA (**Figure 3F-H**).

As depicted in **Figure 3H** and **Figure S3A**-**B**, the overexpression of OSBPL3 in the PK-59 group correlated with high levels of Ki67, while the knockdown group exhibited low Ki67 expression, alongside an opposing trend in CASP3 expression. These results indicated that OSBPL3 promoted the proliferation of PDA cells while simultaneously inhibiting their apoptosis.

Given that enhanced proliferation is often characterized by accelerated G1/S phase transition, we assessed cell cycle dynamics using the GSE155698 single-cell PDA dataset. The OSBPL3 high-expression group exhibited a pronounced enrichment of cells in the G1 and S phases, coupled with a decreased proportion in the G2/M phase, compared to the low-expression group (**Figure S3C**). Similarly, a significant positive correlation was observed between OSBPL3 and various proliferation-related genes, including PCNA (*P* = 1.28e-08, cor = 0.413), MKI67 (*P* <0.0001, cor = 0.558), and CDK1 (*P* <0.0001, cor = 0.556) (**Figure S3D**). Flow cytometry corroborated these findings: in CFPAC-1 cells, OSBPL3 knockdown significantly decreased the G1/S ratio while increasing the proportion of cells in the G2/M phase (*P* < 0.05), whereas OSBPL3 overexpression in PANC-1 cells substantially increased the fraction of G1 phase cells (**Figure S3E**).

In an orthotopic pancreatic tumor model using Panc02 mouse PDA cells, tumors derived from the OSBPL3 overexpression group exhibited significantly greater weight and volume compared to the control group (*P* < 0.0001; **Figure 3J**), indicating enhanced tumor growth. A total of ten mice were used in this experiment. Due to the fragile nature of pancreatic tissue and the tendency for digestion or degradation during handling, only four representative orthotopic tumors were selected for imaging. The remaining tumor samples were either fixed in formalin, preserved in Trizol, or lysed in extraction buffer for subsequent RNA and protein analyses. Consistent with the observed phenotypes, expression of proliferation-related markers PCNA and MKI67 was markedly elevated in the OSBPL3 overexpression group (**Figure 3K**), collectively supporting a pivotal role for OSBPL3 in promoting pancreatic tumor cell proliferation and *in vivo* tumor progression.

### High OSBPL3 expression correlates with enhanced tumor cell stemness, migration, invasion and metastasis

Numerous studies have established that dysregulated lipid metabolism contributes to the acquisition of tumor stemness 34-36. Given that OSBPL3 is involved in the transport of cholesterol and phosphatidylcholine, we sought to further investigate whether OSBPL3 regulates tumor stemness in PDA 37. We employed the CytoTRACE algorithm to infer the differentiation potential of different cell subpopulations based on gene expression profiles and identify progenitor cells. UMAP dimensionality reduction revealed a differentiation hierarchy, with metaplastic cells displaying the highest stemness, followed by ductal cells, while other cell types exhibited lower differentiation potential (**Figure S4A**-**B**). Upon stratifying cells according to OSBPL3 expression, we found that, with the exception of macrophages and pericytes, the CytoTRACE scores for the OSBPL3 high-expression groups were consistently higher than those for the low-expression groups across various cell types (**Figure S4C**).

Additionally, a strong positive correlation was observed between OSBPL3 expression and the GSVA scores derived from two stemness-related gene sets (**Figure S4D**; gene signature 1: *R* = 0.557, *P* = 2.64e-16; gene signature 2: *R* = 0.639, *P* = 2.2e-16). Further predictive analyses of tumor stem cells provided additional corroborative evidence (**Figure S4E**). Examining the proportions of OSBPL3 high- and low-expression cells in three epithelial populations revealed that in metaplastic, ductal, and acinar cells, a higher proportion of OSBPL3 high-expression cells was observed among the CD133⁺/CD44⁺ double-positive cells compared to the negative cell subsets (**Figure S4F**).

**Figure 4:**
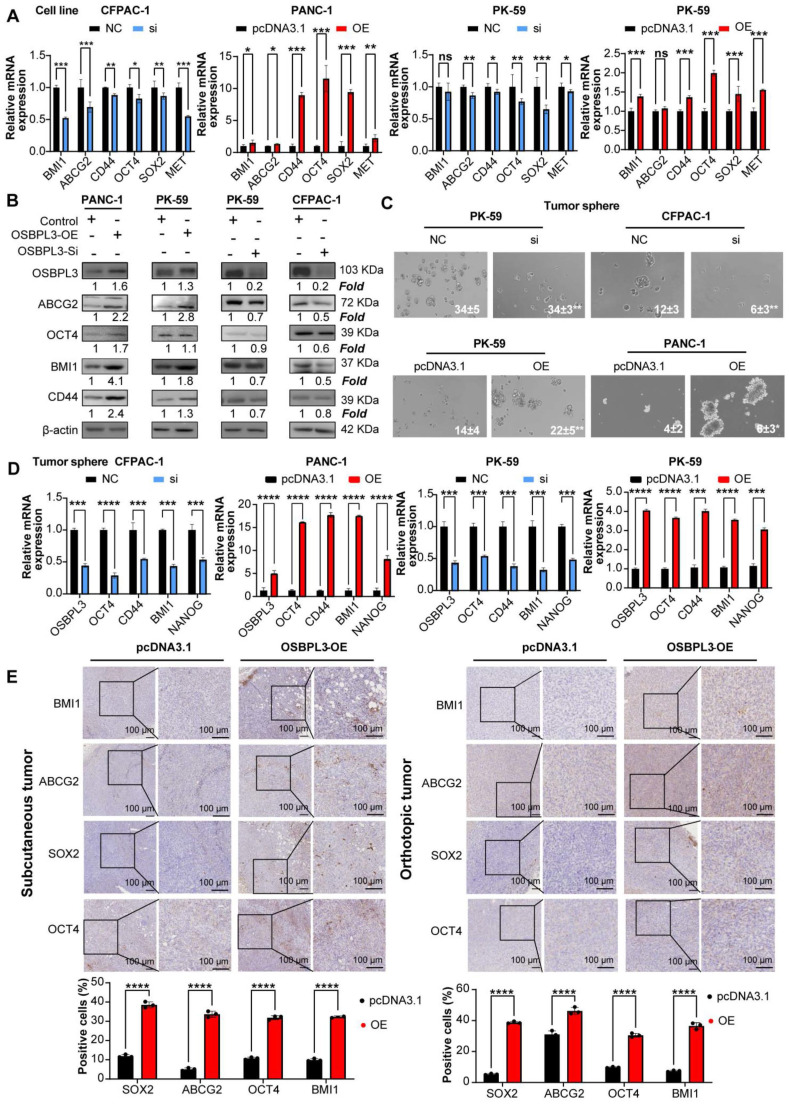
** OSBPL3 promotes the increase of tumor stemness in PDA.** (**A**) qRT-PCR analysis of tumor stemness gene expression in CFPAC-1 and PK-59 cells following OSBPL3 knockdown and in PK-59 and PANC-1 cells following OSBPL3 overexpression (n = 3). (**B**) Western blot analysis of tumor stemness gene expression following OSBPL3 knockdown or overexpression in four different cell lines. (**C**) Tumorsphere formation assay showing changes in stem cell sphere-forming ability following alterations in OSBPL3 expression in PDA cell lines (n = 3). Representative images of tumorspheres are shown. (**D**) qRT-PCR analysis of tumor stemness gene expression in tumorspheres formed in CFPAC-1 and PK-59 cells with OSBPL3 knockdown and in PK-59 and PANC-1 cells with OSBPL3 overexpression (n = 3). (**E**) IHC staining of stemness-related genes in tumors from control and OSBPL3 overexpression groups after constructing subcutaneous tumor and orthotopic tumor models in Panc02 cells. Magnification: 20x and 40x. Statistical analysis of IHC staining images between groups shown as a bar chart. Non-paired Student's t-test, *P < 0.05, **P < 0.01, ***P < 0.001, ****P < 0.0001.

To verify the promoting effect of OSBPL3 on tumor stemness in PDA, we performed both *in vivo* and *in vitro* experiments. As shown in **Figure 4A**-**B**, knockdown of OSBPL3 resulted in decreased levels of ABCG2, OCT4 and BMI1, whereas OSBPL3 overexpression led to increased levels of these proteins relative to β-actin. Tumorsphere formation assays further corroborated these results, showing a significant reduction in tumorsphere size and number in OSBPL3 knockdown CFPAC-1 and PK-59 cell lines, indicating a loss of tumorigenic potential. In contrast, OSBPL3 overexpression in PK-59 and PANC-1 cells resulted in an increased number and size of tumorspheres, suggesting that OSBPL3 promotes the tumorigenic capacity of PDA cells (**Figure 4C**). Notably, we observed that knockdown or overexpression of OSBPL3 led to significant changes in the expression of stemness-associated genes across all cell lines (**Figure 4D**). Immunohistochemistry of subcutaneous and orthotopic tumors from mice further demonstrated increased expression of stemness-associated genes upon OSBPL3 overexpression (**Figure 4E**). These results collectively suggest that OSBPL3 is a key regulator of tumor stemness in PDA.

**Figure 5:**
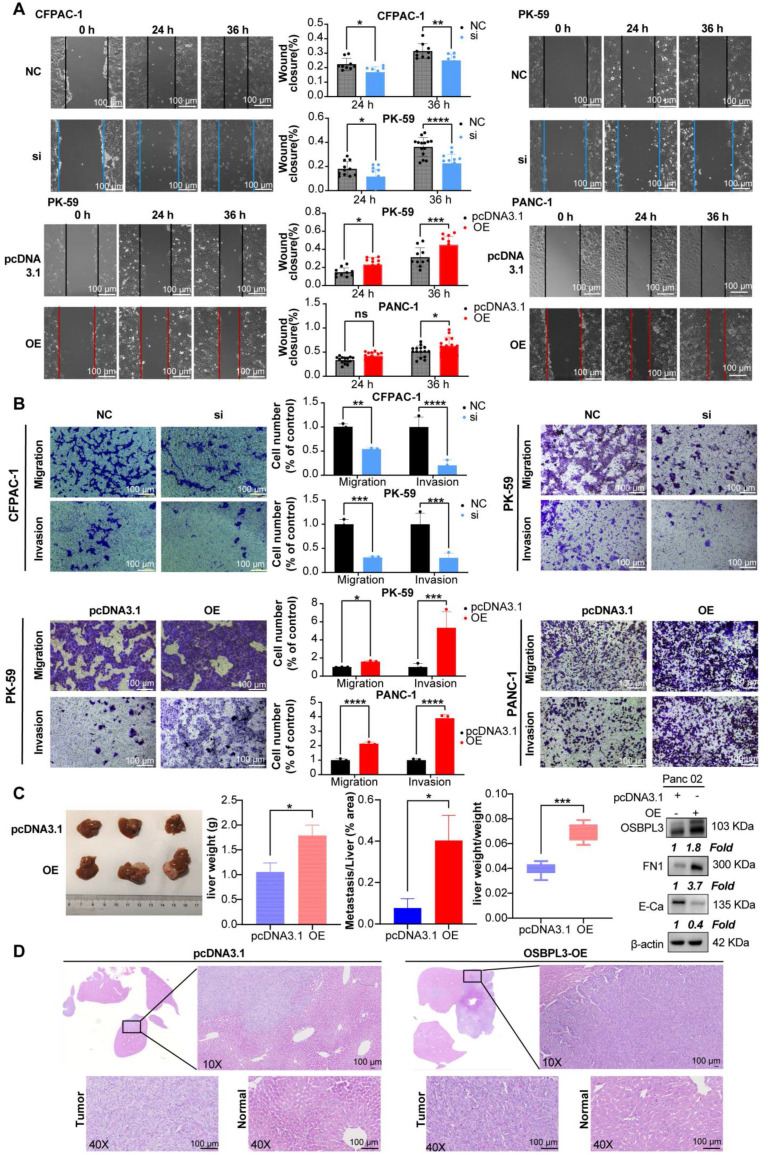
** High expression of OSBPL3 promotes PDA cell migration and invasion.** (**A**) Scratch assay evaluating cell migration rates at 0, 24 and 36 h time points before and after OSBPL3 expression knockdown or overexpression in human PDA cells. Representative images of the two groups at different time points are shown, with the gray group representing the NC control and the blue group representing the siRNA knockdown. The bar chart displays the statistical analysis of cell migration rates at the 24 and 36 h time points. (**B**) Transwell migration and invasion assays showing random representative images of cell migration and invasion after OSBPL3 knockdown or overexpression in PDA cell lines. Magnification: 10x. The bar chart shows migration and invasion rates for different OSBPL3 expression groups. (**C**) Images of liver metastatic tumors from euthanized mice, with the upper row representing the pcDNA3.1 control group and the lower row representing the Osbpl3 overexpression group. Bar charts show statistical analysis of liver weight, the ratio of liver weight to body weight, and the ratio of metastatic area to total liver area. Representative Western blot images showing FN1 and E-cadherin expression in control and OSBPL3-overexpressing group*.* Non-paired Student's t-test, *P < 0.05, **P < 0.01, ***P < 0.001, ****P < 0.0001. E-Ca: E-cadherin. (**D**) Representative H&E staining images of liver metastatic tumors from the pcDNA3.1 control and Osbpl3 overexpression groups. Magnification: 2.5x, 10x and 40x.

Because the elevation of tumor stemness is associated with increased invasiveness and migratory capabilities of tumors, we conducted wound healing assays and Transwell migration experiments using the CFPAC-1 and PK-59 cell lines. Compared to the control, the migration ability was significantly reduced following OSBPL3 knockdown, with area analysis performed via ImageJ indicating statistical differences at both the 24- and 36-h time points (**Figure 5A**). In contrast, the overexpression of OSBPL3 in the two cell lines showed an opposite trend. Transwell migration experiments across different cell lines further confirmed that OSBPL3 promotes PDA migration (**Figure 5B**). Given the elevated expression of OSBPL3 in metastatic tumors, we simulated the invasion of tumor cells by adding matrigel to the upper chamber of the Transwell assay. As shown in **Figure 5B**, the results indicated that the invasion ability of the OSBPL3 overexpression group was significantly enhanced compared to the control group, while the invasion ability of the OSBPL3 knockdown group was notably reduced. Therefore, OSBPL3 may play an important role in PDA metastasis.

Then, we constructed a Panc02 mouse PDA liver metastasis model with OSBPL3 overexpression to study the role of OSBPL3 in liver metastasis* in vivo*. **Figure 5C** displayed representative photographs (n = 3) of the liver from the control and overexpression groups. The OSBPL3 overexpression group exhibited significantly higher liver lesions, liver weight (*P* = 0.017), and the ratio of liver weight to mouse body weight (*P* = 0. 00092) than that in the control (n = 10) (**Figure 5C**). Histological examination of liver samples stained with HE revealed that the percentage of liver metastatic lesions in the OSBPL3 overexpression group (*P* = 0.011) was significantly greater than that in the control (**Figure 5D**). Western blot analysis revealed differential expression of epithelial-mesenchymal transition (EMT) markers between the two groups. Specifically, FN1 (Fibronectin) was upregulated, while E-cadherin was downregulated in the OSBPL3-overexpressing group, indicating EMT activation (**Figure 5D**).

### OSBPL3 affects chemotherapy sensitivity to oxaliplatin in pancreatic cancer

Both oxaliplatin and irinotecan are essential components of the first-line chemotherapy regimen FOLFIRI-NOX for PDA. The correlation between the IC50 values of oxaliplatin and OSBPL3 expression was highly significant (*P* = 3.01e-7, *R* = 0.368) (**Figure S5A**).

**Figure 6:**
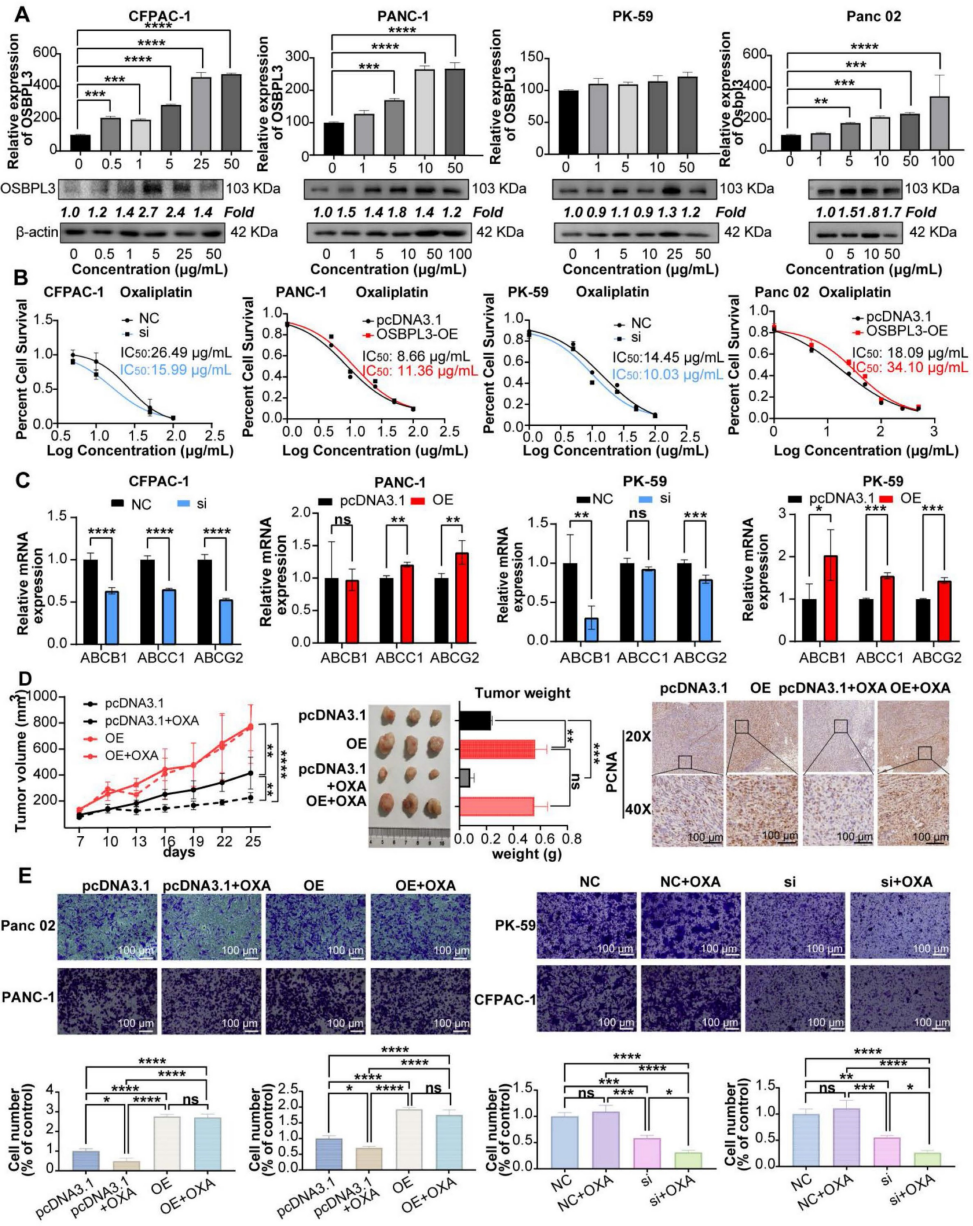
** OSBPL3 overexpression contributes to oxaliplatin resistance in PDA.** (**A**) qRT-PCR and Western blot analysis of OSBPL3 expression relative to the internal control β-actin in different cell lines following treatment with varying concentrations of oxaliplatin. (**B**) Changes in the half-maximal inhibitory concentration (IC50) of oxaliplatin following OSBPL3 knockdown or overexpression in CFPAC-1, PANC-1, Panc02 and PK-59 cell lines. Black represents the control group, red represents the OSBPL3 overexpression (OE) group, and blue represents the OSBPL3 knockdown (Si) group. (**C**) mRNA expression levels of the multidrug resistance genes ABCB1, ABCC1, and ABCG2 in CFPAC-1, PANC-1 and PK-59 PDA cell lines, compared to the NC control group, following OSBPL3 knockdown or overexpression. Non-paired Student's t-test, *P < 0.05, **P < 0.01, ***P < 0.001, ****P < 0.0001. (**D**) Tumor volume measurement started on day 7 after subcutaneous tumor inoculation. On day 10, oxaliplatin was administered by intraperitoneal injection, followed by tumor volume measurements every 3 days (n = 10). The data are presented as a line graph. Representative images of subcutaneous tumors in the normal control (pcDNA3.1) group, OSBPL3 overexpression (OE) group, and their respective oxaliplatin-treated groups (pcDNA3.1+OXA and OE+OXA). Statistical analysis of tumor weight for the above-mentioned groups. Representative images of IHC staining for the cell proliferation marker PCNA in the four groups, at 20x and 40x magnification. Statistical analysis of the number of positive cells in PCNA immunohistochemistry is shown in the bar chart. Non-paired Student's t-test, *P < 0.05, **P < 0.01, ***P < 0.001, ****P < 0.0001. (**E**) Representative images from the Transwell migration assay showing cell migration in NC control, NC oxaliplatin treatment, OSBPL3 knockdown, and OSBPL3 knockdown + oxaliplatin treatment in Panc02, PANC-1, PK-59, and CFPAC-1 cell lines. Magnification: 10x. The bar chart shows statistical analysis of migration rates for each group. ANOVA and multiple t-tests were performed. ns indicates no significant difference, *P < 0.05, **P < 0.01, ***P < 0.001, ****P < 0.0001.

As shown in **Figure 6A**, both mRNA and protein levels of OSBPL3 increased in response to varying concentrations of oxaliplatin. Notably, in the PANC-1, Panc02 and CFPAC-1 cell lines, OSBPL3 expression levels rose significantly with increasing drug concentrations. In the PK-59 cell line, although the increase in OSBPL3 expression with concentration was less pronounced, it remained higher than in the untreated group.

We measured cell viability in response to varying oxaliplatin concentrations using CCK8 assays, calculating the half maximal inhibitory concentration (IC_50_) for each cell line. OSBPL3 knockdown led to a decrease in IC_50_ values, indicating increased oxaliplatin sensitivity. Specifically, in the CFPAC-1 cell line, the IC_50_ decreased from 26.49 μg/mL in the control (NC) group to 15.99 μg/mL in the knockdown (si) group. Similarly, in the PK-59 cell line, IC_50_ dropped from 14.45 μg/mL in the control to 10.03 μg/mL in the knockdown group (**Figure 6B**). Conversely, in the PANC-1 cell line, overexpression of OSBPL3 increased the IC_50_ from 8.66 to 11.36 μg/mL, and similar results were observed in the mouse Pan02 cell line (**Figure 6B**). OSBPL3's impact on multi-drug resistance was further supported by analyzing the expression of ABC transporters, known to facilitate chemoresistance by expelling chemotherapy drugs. In CFPAC-1 and PK-59 cells, OSBPL3 knockdown reduced the expression of multi-drug resistance genes ABCB1, ABCC1, and ABCG2, while OSBPL3 overexpression increased their expression (**Figure 6C**). These results affirm that OSBPL3 modulates the expression of drug-resistance genes, suggesting its role in PDA chemoresistance.

To investigate the effects of OSBPL3 expression on tumor growth and oxaliplatin resistance* in vivo*, we used a mouse subcutaneous tumor model. As shown in **Figure 6D**, OSBPL3 overexpression significantly increased tumor size, weight, and growth rate, regardless of oxaliplatin treatment. In the pcDNA3.1 control group, oxaliplatin treatment moderately increased tumor growth compared to the untreated control. However, in the OSBPL3 overexpression group, oxaliplatin treatment did not significantly affect tumor growth, suggesting resistance. Immunohistochemical staining for the proliferation marker PCNA further confirmed significant differences among the groups (**Figure 6D**). In the Panc02 mouse cell line, we generated an OSBPL3 overexpression model (OE) and compared it with the control (pcDNA3.1) in wound healing assays following oxaliplatin treatment. At both 12 and 24 h, OSBPL3 overexpression (OE+OXA) led to enhanced migration compared to the control group (pcDNA3.1+OXA), indicating oxaliplatin resistance (**Figure S6C**). Similar trends were observed in Transwell migration assays for both human and mouse cell lines, supporting the role of OSBPL3 in promoting PDA cell migration and resistance to oxaliplatin (**Figure 6E**).

To assess whether OSBPL3 directly interacts with oxaliplatin, we performed molecular docking simulations. Multiple potential binding residues were identified, including GLY-704 and ASN-721 (**Figure S5B**). However, the predicted binding affinities were relatively weak (all < -5.7 kcal/mol), suggesting that OSBPL3 is unlikely to mediate chemoresistance through direct physical interaction with oxaliplatin. Accordingly, we shifted our focus to alternative mechanisms, including OSBPL3-driven modulation of drug metabolism, regulation of drug efflux or uptake transporters, and activation of key survival pathways involved in chemoresistance. These findings collectively demonstrate that OSBPL3 promotes chemoresistance in PDA, particularly influencing sensitivity to oxaliplatin. This underscores OSBPL3 as a potential therapeutic target for overcoming chemoresistance in PDA.

### OSBPL3 regulates the malignant behaviors of PDA by activating oncogenic pathways such as NOTCH

Transcriptome sequencing was performed on samples from CFPAC-1 cells treated with control siRNA (NC) and OSBPL3_siRNA (knockdown). GSEA analysis of the sequencing data revealed that the Wnt, Notch, Hippo, and Hedgehog signaling pathways exhibited lower enrichment scores in the OSBPL3_siRNA group than that in the NC group (**Figure S6A**). Similarly, genes upregulated in epithelial cells with high OSBPL3 expression were significantly enriched in the Notch signaling pathway (**Figure S6B-C**). GSVA analyses across multiple databases (KEGG, PID, GO, Reactome) further confirmed that tumor stemness-related pathways, including Notch, were significantly enriched in high OSBPL3 expression group (**Figure S6D**). These findings suggest that OSBPL3 play an important role in activating the Notch signaling pathway in PDA.

**Figure 7:**
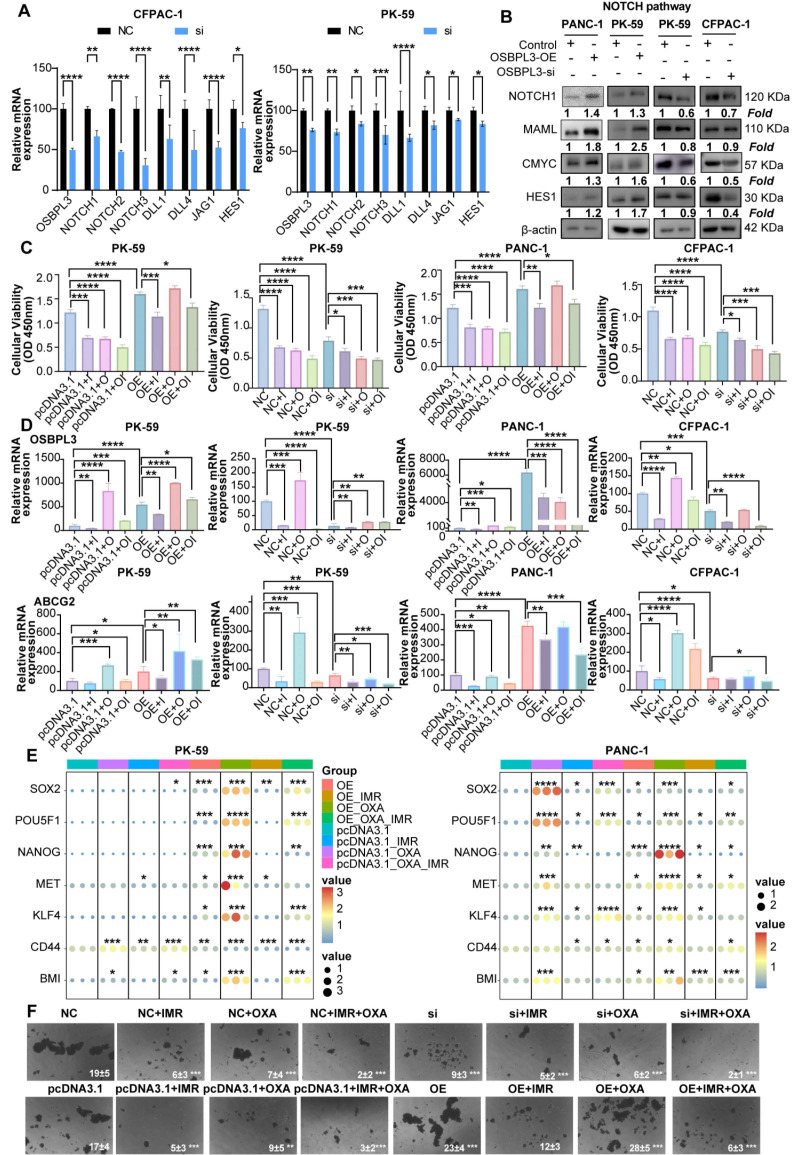
** Inhibition of the NOTCH pathway may reverse the increase in tumor stemness and oxaliplatin resistance induced by OSBPL3 overexpression.** (**A**) qRT-PCR and (**B**) Western blot analyses were performed to assess the expression levels of key NOTCH pathway genes in PDA cells before and after OSBPL3 knockdown or overexpression. (**C**) CCK-8 assays were conducted to evaluate cell viability in various treatment groups: untreated controls (NC and pcDNA3.1 groups), NOTCH pathway inhibitor (IMR) treatment (NC+IMR and pcDNA3.1+IMR groups), oxaliplatin treatment (NC+OXA and pcDNA3.1+OXA groups), and combined IMR and oxaliplatin treatment (NC+IMR+OXA and pcDNA3.1+IMR+OXA groups), along with the corresponding OSBPL3 knockdown or overexpression groups. (**D**) qRT-PCR analysis was performed to examine the expression of OSBPL3 and ABCG2 in cells before and after treatment with oxaliplatin and/or IMR. (**E**) mRNA expression levels of tumor stemness-related genes were evaluated in PK-59 and PANC-1 cells following OSBPL3 overexpression, IMR and oxaliplatin treatments. Relative expression levels are represented by the size and color of the bubbles. (**F**) Tumorsphere formation assay under different treatment conditions. Representative images of tumorspheres formed by PDAC cells transfected with either negative control (NC/pcDNA3.1), OSBPL3 knocwdown (Si) or OSBPL3 overexpression (OE) vectors and treated with IMR (a NOTCH inhibitor), oxaliplatin (OXA), or their combination. Data are presented as mean ± SD, *n = 3 independent experiments*, Non-paired Student's t-test, *P < 0.05, **P < 0.01, ***P < 0.001, ****P < 0.0001.

To verify the role of OSBPL3 in the NOTCH pathway, we performed western blot and qRT-PCR analyses and found that the expression levels of key Notch signaling pathway genes were positively correlated with OSBPL3 expression (**Figure 7A**-**B**). Cell communication analyses centered on the Notch signaling pathway revealed that ductal cells with high OSBPL3 expression exhibited significantly more intercellular communication than those with low expression (**Figure S6E**). High OSBPL3-expressing ductal cells primarily interacted with pericytes, fibroblasts, and monocyte-macrophages via Notch-related pathways (**Figure S7F**-**G**). Furthermore, the expression distribution of Notch pathway components demonstrated that JAG1 and NOTCH2 exhibited the highest communication scores in OSBPL3 high-expressing ductal cells (**Figure S7H**). These findings suggested that changes in OSBPL3 expression influenced many important regulatory pathways like NOTCH.

We further investigated whether NOTCH pathway inhibition could modulate OSBPL3-induced chemoresistance and tumor stemness. OSBPL3 was knocked down in CFPAC-1 and PK-59 cells, and cells were subsequently treated with oxaliplatin (OXA), the NOTCH inhibitor IMR, or their combination. As shown in **Figure 7C**, in control cells, both OXA and IMR monotherapies significantly reduced cell viability, and their combination exerted an additive inhibitory effect. However, in OSBPL3-overexpressing cells, OXA monotherapy paradoxically promoted cell proliferation, indicating the development of drug resistance. Notably, the addition of IMR restored chemosensitivity and significantly reduced cell viability in OSBPL3-overexpressing cells, suggesting that NOTCH inhibition could overcome OSBPL3-mediated resistance. Consistent with these findings, qRT-PCR analysis revealed that IMR treatment markedly reduced the expression of OSBPL3, the multidrug resistance gene ABCG2, and several stemness-associated markers (**Figure 7D**-**E**). In contrast, OXA monotherapy upregulated the expression of several of these genes, particularly in the context of OSBPL3 overexpression. This upregulation was effectively reversed by the addition of IMR, paralleling the cell viability trends. These observations suggest that sublethal oxaliplatin exposure may enhance stem-like features, a phenomenon exacerbated by OSBPL3 and mitigated through NOTCH inhibition. To functionally validate the impact on tumor stemness, we conducted tumorsphere formation assays under various treatment conditions (**Figure 7F**). Oxaliplatin was applied at slightly above its IC50 to mimic therapeutic pressure. OSBPL3 overexpression significantly increased both the number and size of tumorspheres, reflecting enhanced self-renewal capacity. Oxaliplatin treatment alone also promoted tumorsphere formation, particularly in OSBPL3-overexpressing cells, further supporting the idea that chemotherapy may enrich cancer stem-like populations. Importantly, treatment with IMR, either alone or in combination with oxaliplatin, markedly suppressed tumorsphere formation. The OE+IMR and OE+IMR+OXA groups showed significantly fewer and smaller spheres compared to the OE and OE+OXA groups, highlighting the critical role of NOTCH signaling in sustaining OSBPL3-driven stemness under chemotherapeutic stress.

### OSBPL3 regulates NOTCH pathway and the malignant behavior of PDA by mediating sterol synthesis

Numerous studies have suggested that OSBPL3 plays a pivotal role in steroid and lipid metabolism. To comprehensively investigate the relationship between OSBPL3 and lipid-associated metabolic pathways, we integrated human and murine single-cell RNA-seq datasets of pancreatic cancer and performed pathway enrichment analyses using irGSEA and scMetabolism algorithms. The gene set of steroid-related pathways is shown in **Table S4**. These analyses revealed that high OSBPL3 expression was associated with enhanced activity in lipid-related metabolic pathways, particularly fatty acid synthesis and cholesterol biosynthesis (**Figure S7A-E**).

**Figure 8:**
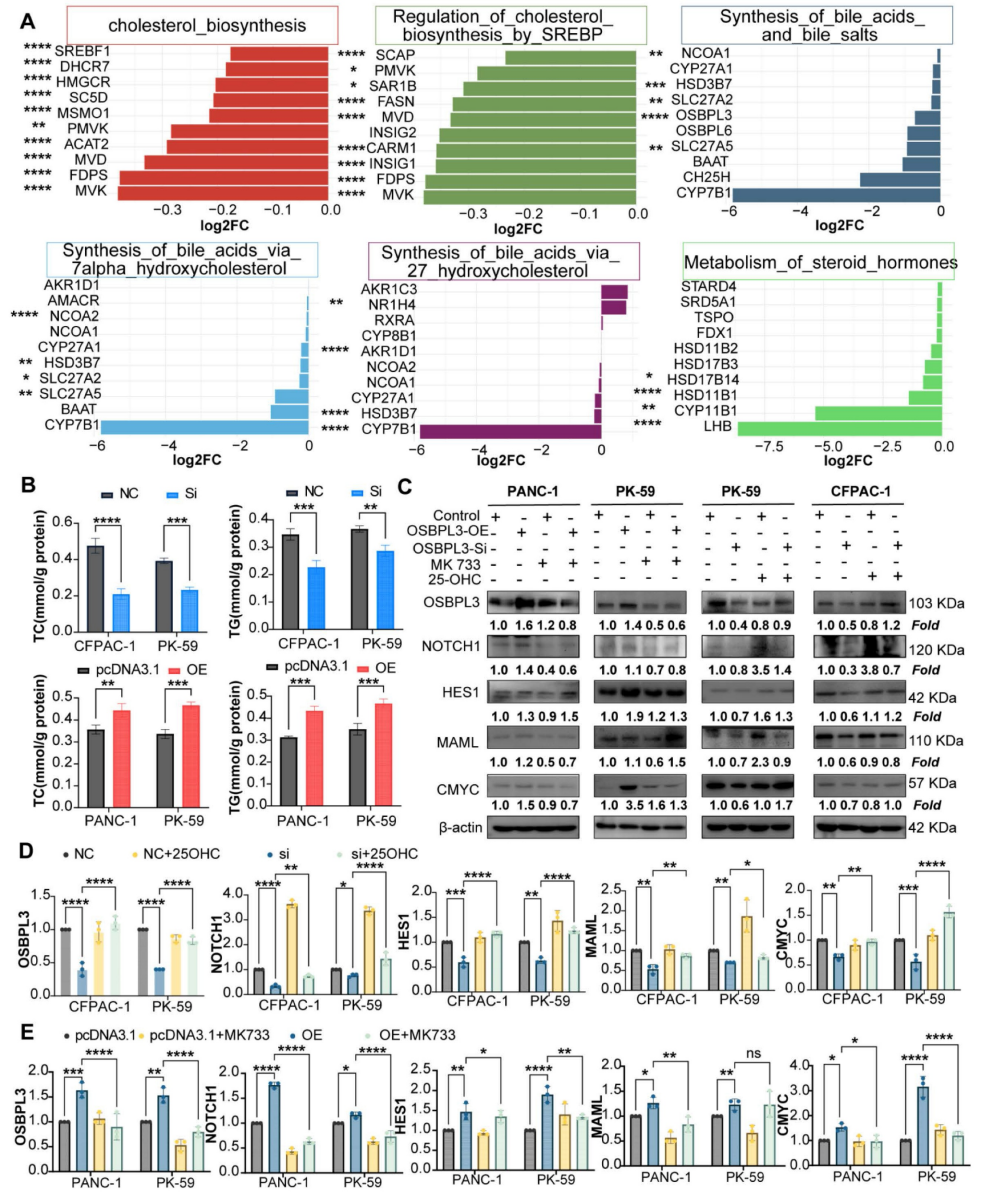
** OSBPL3 regulates NOTCH signaling pathway activity in pancreatic cancer cells via cholesterol metabolism.** (**A**) Differential expression of cholesterol related pathways and genes before and after OSBPL3 knockdown, with the x-axis representing key genes involved in cholesterol synthesis and the y-axis displaying the log2 fold change (log2FC) of gene expression between the two groups. Non-paired Student's t-test, *P < 0.05, **P < 0.01, ***P < 0.001, ****P < 0.0001. (**B**) qRT-PCR analyses were performed to assess the expression levels of key cholesterol related genes in PDA cells before and after OSBPL3 knockdown or overexpression (n = 3). (**C**) Western blot analysis showing that simvastatin (15 nM, 24 h) treatment reduced NOTCH signaling activity in OSBPL3-overexpressing cells, while 25-hydroxycholesterol (25-OHC, 2.5 μM, 24 h) rescued NOTCH activation in OSBPL3-knockdown cells. Densitometry values are normalized to control. β-actin served as the loading control. Simvastatin: MK733; 25-hydroxycholesterol:2-OHC. (**D**) Quantitative analysis of the Western blot results shown in (C) for OSBPL3-knockdown cells. Data are presented as mean ± SD from three independent experiments. Statistical significance was determined by one-way ANOVA followed by Tukey's post-hoc test. *P < 0.05, **P < 0.01, ***P < 0.001, ****P < 0.0001. (**E**) Quantitative analysis of the Western blot results shown in (C) for OSBPL3-overexpressing cells. Data are presented as mean ± SD from three independent experiments. Statistical significance was determined by one-way ANOVA followed by Tukey's post-hoc test. *P < 0.05, **P < 0.01, ***P < 0.001, ****P < 0.0001.

Protein-protein interaction network analysis further suggested a potential crosstalk between OSBPL3, the NOTCH signaling pathway, and key cholesterol biosynthesis genes (**Figure S7F**). Supporting this finding, transcriptome sequencing of CFPAC-1 cells before and after OSBPL3 knockdown demonstrated reduced expression of multiple cholesterol biosynthesis genes and related signaling pathways (**Figure 8A, Figure S8A**).

To further confirm the regulatory effect of OSBPL3 on lipid metabolism, we measured intracellular total cholesterol (TC) and triglyceride (TG) levels in pancreatic cancer cells following OSBPL3 overexpression or knockdown. As shown in Figure 8B, OSBPL3 overexpression in CFPAC-1 and PK-59 cells resulted in a significant increase in both TC and TG contents compared with control cells, while OSBPL3 silencing in PANC-1 cells led to a marked reduction in these lipid metabolites.

To validate these findings at the metabolite level, we analyzed matched transcriptomic and mass spectrometry-based lipidomic data from 20 pancreatic ductal adenocarcinoma (PDA) organoid samples. Partial least squares discriminant analysis (PLS-DA) revealed distinct lipid metabolic profiles between OSBPL3-high and -low groups. Principal component analysis (PCA) also showed a trend toward spatial separation, indicating molecular heterogeneity between the groups; however, due to limited sample size and within-group variability, the 95% confidence intervals partially overlapped, and the separation did not reach statistical significance (**Figure S7G**).

Correlation analysis identified cholesteryl ester CE (22:6) as the metabolite most strongly associated with OSBPL3 expression (correlation coefficient *R* = 0.42, *P* = 0.062), suggesting a potential regulatory relationship despite the lack of statistical significance (**Figure S7F**). These results collectively underscore the potential role of OSBPL3 in modulating cholesterol synthesis and broader lipid metabolic reprogramming in pancreatic cancer (**Figure S7H**).

To further investigate whether OSBPL3 regulates NOTCH activation through cholesterol metabolism, we treated OSBPL3-overexpressing cells with simvastatin, a cholesterol synthesis inhibitor, and OSBPL3-knockdown cells with 25-hydroxycholesterol (25-HC), an oxysterol that restores intracellular cholesterol 38-40. Simvastatin treatment notably suppressed NOTCH signaling activity in OSBPL3-overexpressing cells, while 25-HC partially rescued the reduced expression of NICD and HES1 in OSBPL3-knockdown cells (**Figure 8C**-**D, Figure S8B**). These findings suggest that OSBPL3 activates the NOTCH signaling pathway at least partially through modulation of intracellular cholesterol homeostasis.

Recent findings indicated that Perhexiline maleate can downregulate SREBF2 expression, thereby inhibiting cholesterol metabolism and reversing metabolic reprogramming induced by KRAS mutations in PDA organoids. To investigate the effects of inhibiting key cholesterol synthesis genes on tumor stemness and malignant behaviors in PDA, we utilized single-cell transcriptomic data to examine the effects of SREBF2 inhibition on tumor stemness. We analyzed samples from PDA organoid models to compare the control (KPSC:* Pdx1-Cre; LSL-Kras^G12D/+^; LSL-Tp53^R172H/+^; Smad4^-/-^*) with those treated with Perhexiline maleate (KPSCP:* Pdx1-Cre; LSL-Kras^G12D/+^; LSL-Tp53^R172H/+^; Smad4^-/-^* with Perhexiline maleate) and found a decrease in expression of Osbpl3, Srebf1, and Srebf2 in KPSCP group (**Figure S9A**). Stemness gene set scoring revealed upregulation in KPSC and downregulation in KPSCP (**Figure S9B**-**C**).

Then, we turned to another single-cell transcriptomic dataset from KPC mice with conditional inactivation of the cholesterol synthesis enzyme Nsdhl. Upon performing UCell scoring on HALLMARK gene sets related to oncogenic pathways, we found notable reductions in scores for pathways associated with NOTCH in the KPCN group. The results suggested that inhibiting cholesterol synthesis may simultaneously suppress PDA metastasis and progression (**Figure S9D**) and decreased the scores for cholesterol homeostasis in the KPCN group (**Figure S9E**).

We further analyzed common tumor stemness genes in both groups before and after Nsdhl inactivation. Genes such as Cd44, Abcg2, Klf4, Met and Bmi1 exhibited significantly lower expression in the KPCN group (**Figure S9F**). Using five different algorithms, including AUCell and Ucell, we evaluated stemness gene sets across both groups. The KPCN group consistently displayed significantly lower stemness scores (**Figure S9G**), and the violin plot confirmed these findings of the AUCell score for Shats-iPSC stemness gene set (**Figure S9H**).

## Discussion

Oxysterol-binding protein-like 3 (OSBPL3), a member of the oxysterol-binding protein-related family, is abnormally overexpressed in multiple human malignancies and plays critical roles in lipid transport, intracellular signaling, and membrane dynamics 41. In this study, we comprehensively investigated the expression, function, and underlying mechanisms of OSBPL3 in pancreatic ductal adenocarcinoma (PDA) using multi-omics data integration and extensive experimental validation. Our analyses of single-cell transcriptomic datasets from both human PDA and the KC mouse model of pancreatic carcinogenesis revealed a consistent upregulation of OSBPL3 during PDA progression. These findings were further confirmed by *in vivo* models of pancreatitis and PDA, supporting the notion that OSBPL3 is involved in the early and sustained phases of tumor development 22,42,43. Our results established the pivotal role of OSBPL3 in PDA development and progression. Moreover, subgroup and correlation analyses combining transcriptomic and clinical data revealed that patients with high OSBPL3 expression had shorter overall survival. OSBPL3 expression was also associated with tumor staging and minimal residual disease in PDA patients. These findings indicated that OSBPL3 functions as a critical oncogene in PDA and serves as an important biomarker of poor prognosis and a potential therapeutic target. OSBPL3 may contribute to clinical stratification and personalized treatment in PDA, providing new avenues for prognosis assessment and precision treatment.

Functional assays demonstrated that OSBPL3 promotes PDA cell proliferation, migration, invasion, and epithelial-to-mesenchymal transition (EMT). Although the pro-proliferative role of OSBPL3 in PDA has not yet been reported, Shan *et al*. found that OSBPL3 upregulation promotes proliferation while inhibiting apoptosis and pyroptosis in endometrial cancer cells 44. Similarly, Njeru *et al*. suggested that OSBPL3 mutations may influence tumor cell proliferation and survival by altering lipid metabolism. Zhang and colleagues analyzed samples from 92 colorectal cancer patients and discovered a significant correlation between OSBPL3 expression and Ki67 levels in colorectal cancer tissues 21. Our results from single-cell transcriptomic analyses, transwell invasion and migration assays, and a PDA liver metastasis mouse model revealed that aberrant OSBPL3 expression enhances the migratory and invasive capabilities of PDA cells, induces epithelial-to-mesenchymal transition (EMT), and thereby promotes metastasis. These findings indicate that OSBPL3 can augment the migration and invasion of PDA cells. While studies on OSBPL3's role in tumor metastasis remain limited, the pro-metastatic effects of other OSBPL family members have been confirmed in several studies 45,46.

One of the most striking findings from this study is the role of OSBPL3 in enhancing tumor cell stemness and chemoresistance. One of the most striking findings from this study is the role of OSBPL3 in enhancing tumor cell stemness and chemoresistance. High OSBPL3 expression correlated with stemness scores derived from CytoTRACE and mRNA expression signatures, and its overexpression enriched PDA cells with cancer stem cell-like properties. These cells exhibited increased resistance to oxaliplatin, a commonly used chemotherapeutic agent in PDA, suggesting that OSBPL3 contributes to adaptive survival mechanisms under therapeutic stress. Although oxaliplatin alone had limited and transient efficacy *in vivo*, combination treatment with IMR (a NOTCH inhibitor) reversed OSBPL3-induced chemoresistance, implicating the cholesterol-NOTCH axis in OSBPL3-mediated effects.

Mechanistically, we identified a functional link between OSBPL3, cholesterol metabolic reprogramming, and NOTCH signaling. Transcriptomic and pathway enrichment analyses revealed elevated NOTCH pathway activity in OSBPL3-overexpressing cells. Pharmacologic inhibition of NOTCH signaling reduced OSBPL3 expression, reversed chemoresistance, and suppressed cell viability. Furthermore, cholesterol depletion and rescue experiments provided direct evidence that cellular cholesterol levels modulate NOTCH pathway activity. This supports the notion that OSBPL3 may exert its effects, at least in part, through cholesterol-mediated regulation of NOTCH signaling. Previous studies have implicated other OSBPL family members, such as OSBPL5 and ORP2, in NOTCH regulation, suggesting a broader functional convergence between sterol metabolism and oncogenic signaling 46-48.

Further analyses demonstrated that OSBPL3 overexpression enhances cholesterol biosynthesis and uptake. Downregulation of key cholesterol metabolism genes, such as NSDHL and SREBF2, diminished NOTCH activity and stemness-related gene expression, suggesting that OSBPL3 may modulate PDA malignancy via a cholesterol-NOTCH-stemness axis 49. These findings position OSBPL3 as both a metabolic and signaling hub in PDA.

Given OSBPL3's physiological role in cholesterol transport and steroid biosynthesis, systemic inhibition could lead to adverse effects such as hepatic toxicity, endocrine imbalance, or hematopoietic suppression 50,51. To mitigate these concerns, several strategies could be employed. Tumor-specific delivery systems—such as nanoparticles conjugated with PDA-selective ligands—may enhance drug accumulation in tumors while reducing off-target toxicity 52-54. Additionally, transcriptional targeting using tumor-selective promoters or combinatorial treatment regimens may restrict OSBPL3 suppression to malignant cells and improve therapeutic indices 55. Although OSBPL3-selective inhibitors are not yet available, OSW-1 and other compounds targeting OSBP-related pathways have shown low systemic toxicity in preclinical models, offering a promising starting point for drug development 56,57.

Despite these novel insights, several limitations must be acknowledged. First, we did not perform targeted lipidomics or sterol metabolite profiling, which would directly confirm the functional role of OSBPL3 in cholesterol reprogramming. Second, although transcriptomic and pharmacologic evidence support the involvement of the NOTCH pathway, the exact molecular mechanism linking OSBPL3, cholesterol homeostasis, lipid raft remodeling, and NOTCH activation remains to be elucidated. In particular, whether OSBPL3 affects the localization or stability of NOTCH receptors within cholesterol-rich microdomains warrants further exploration. Third, our current study primarily utilized transient overexpression and knockdown models. These approaches may not fully capture the long-term or epigenetic effects of OSBPL3 deregulation. Future research using CRISPR/Cas9-based genome editing could provide more stable and precise modulation of OSBPL3 in PDA models 58. In addition, the majority of our validation experiments were performed in cell lines and murine models. The translation of our findings to human PDA requires validation in large cohorts of clinical specimens with well-annotated treatment and outcome data. To further validate the proposed cholesterol-NOTCH-stemness axis, future studies should focus on the following directions: (1) integrating lipidomic and transcriptomic sequencing in OSBPL3-modulated PDA models; (2) isolating and characterizing lipid rafts in OSBPL3-overexpressing or -knockdown cells to assess NOTCH receptor localization and activation; (3) developing or identifying selective and safe OSBPL3-targeted agents and assessing their efficacy in preclinical models; and (4) validating the diagnostic and prognostic value of OSBPL3 in clinical PDA samples and exploring its relevance in pancreatitis-related carcinogenesis. In summary, our study provides compelling evidence that OSBPL3 is a key driver of PDA progression through its role in lipid metabolic reprogramming, promotion of tumor stemness, and regulation of chemoresistance via the NOTCH pathway. These findings suggest that OSBPL3 is a promising biomarker for risk stratification and a potential therapeutic target for enhancing treatment efficacy in PDA.

## Supplementary Material

Supplementary figures.

Supplementary tables.
