# Supplementary material for: OSBPL3-driven sterol metabolic reprogramming promotes oncogenic signaling and therapeutic resistance in pancreatic cancer
Source: Theranostics. 2026 Feb 18;16(9):4745–67. doi: 10.7150/thno.113637 (PMC12964224; doi:10.7150/thno.113637)
Supplement: Supplementary file 1 — Supplementary figures. [file thnov16p4745s1.pdf]

OSBPL3-Driven Sterol Metabolic Reprogramming Promotes Oncogenic Signaling and Therapeutic Resistance in Pancreatic Cancer

Running title: OSBPL3 in PDA Progression

Qihui Sun[1,3#], Xiaojia Li[1,3#], Qi Zou[1,2,3#], Yang Chen[1,3], Xiaoqi Zhu[1,2,3], Hailin Jiang[1,3], Tingting Jiang[1,3], Fang Wei[1,2,3], Keping Xie[1,2,3*]

[1]Center for Pancreatic Cancer Research, The South China University of Technology School of Medicine, Guangzhou, Guangdong 510006, China

[2]Guangzhou Digestive Disease Center, Guangzhou First People's Hospital and The Second Affiliated Hospital, South China University of Technology School of Medicine, Guangzhou, China

[3]The South China University of Technology Comprehensive Cancer Center, Guangzhou, Guangdong 510006, China

[*]*Correspondence authors*: Keping Xie or Fang Wei, Center for Pancreatic Cancer Research, South China University of Technology School of Medicine, Guangzhou 510006, China; *E-mail*: mcxiekeping@scut.edu.cn.

Disclosure of Potential Conflicts of Interest: The authors declare no conflict of interest.

Financial Support: The work is partly supported by National Natural Science Foundation of China (#82072632) and Guangzhou Municipality Bureau of Science and Technology, Guangzhou, China (#202102010033) and Natural Science Foundation of Guangdong Province, China (#2022A1515012585).

[#]Q. Sun，X. Li and Q. Zou have contributed equally to this work and are considered co-first authors.

Supplementary Figures

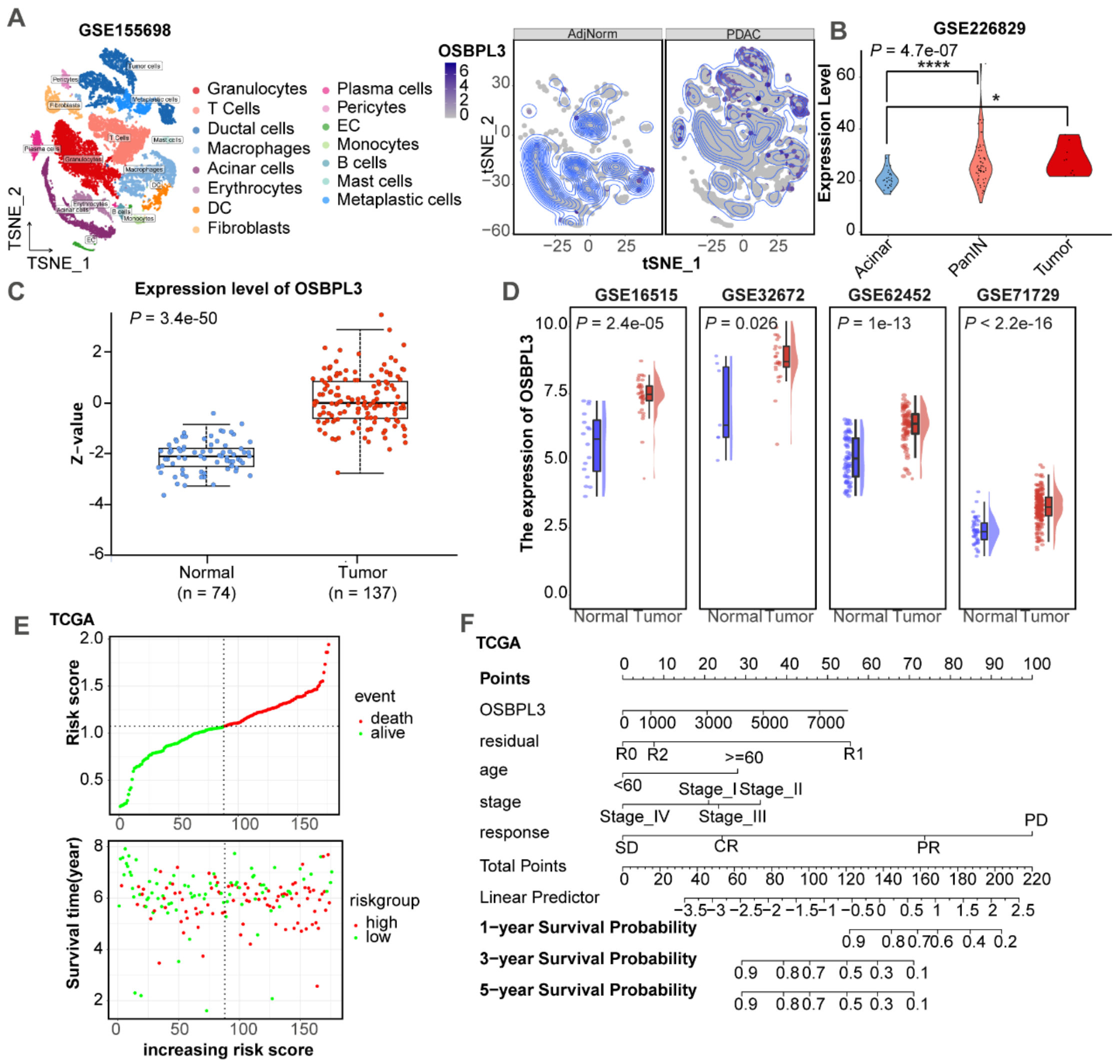
A
GSE155698
TSNE_2
TSNE_1
Granulocytes
T Cells
Ductal cells
Macrophages
Acinar cells
Erythrocytes
DC
Fibroblasts
Plasma cells
Pericytes
EC
Monocytes
B cells
Mast cells
Metaplastic cells
OSBPL3
AdjNorm
PDAC
tSNE_2
tSNE_1
B
GSE226829
P = 4.7e-07
****
*
Expression Level
Acinar
PanIN
Tumor
C
Expression level of OSBPL3
P = 3.4e-50
Z-value
Normal
(n = 74)
Tumor
(n = 137)
D
GSE16515
GSE32672
GSE62452
GSE71729
P = 2.4e-05
P = 0.026
P = 1e-13
P < 2.2e-16
The expression of OSBPL3
Normal Tumor
E
TCGA
Risk score
event
death
alive
Survival time(year)
riskgroup
high
low
increasing risk score
F
TCGA
Points
OSBPL3
residual
age
stage
response
Total Points
Linear Predictor
1-year Survival Probability
3-year Survival Probability
5-year Survival Probability
R0 R2 >=60 R1
<60
Stage_I Stage_II
Stage_IV Stage_III
SD CR PR PD

Figure S1. High expression of OSBPL3 in pancreatic cancer and precancerous lesions correlates with poor prognosis in human PDA Samples

(**A**) UMAP plot showing the clustering of 15 cell groups derived from single-cell transcriptomic data of 16 PDA tissues and 3 adjacent normal tissues from the GSE155698 dataset. The expression density map reveals significantly higher OSBPL3 expression in tumor tissues, predominantly within tumor cells.

(**B**) OSBPL3 expression across acinar cells, PanIN lesions, and tumor cell regions in the GSE226829 dataset. Cells from different regions are represented in distinct colors.

(**C**) Boxplot showing the protein expression of OSBPL3 in PDA and adjacent normal tissues from the CPTAC dataset in the UALCAN database (n = 211).

(**D**) OSBPL3 expression levels in PDA tissues compared to normal control tissues across transcriptomic datasets (GSE16515, GSE32672, GSE62452, GSE71729) from the GEO database. Blue: normal control group; Red: tumor tissue group.

(**E**) Risk factor density map showing OSBPL3 expression in PDA.

(**F**) Nomogram illustrating 1-, 3- and 5-year survival predictions based on OSBPL3 expression levels, age, stage and other prognostic factors.

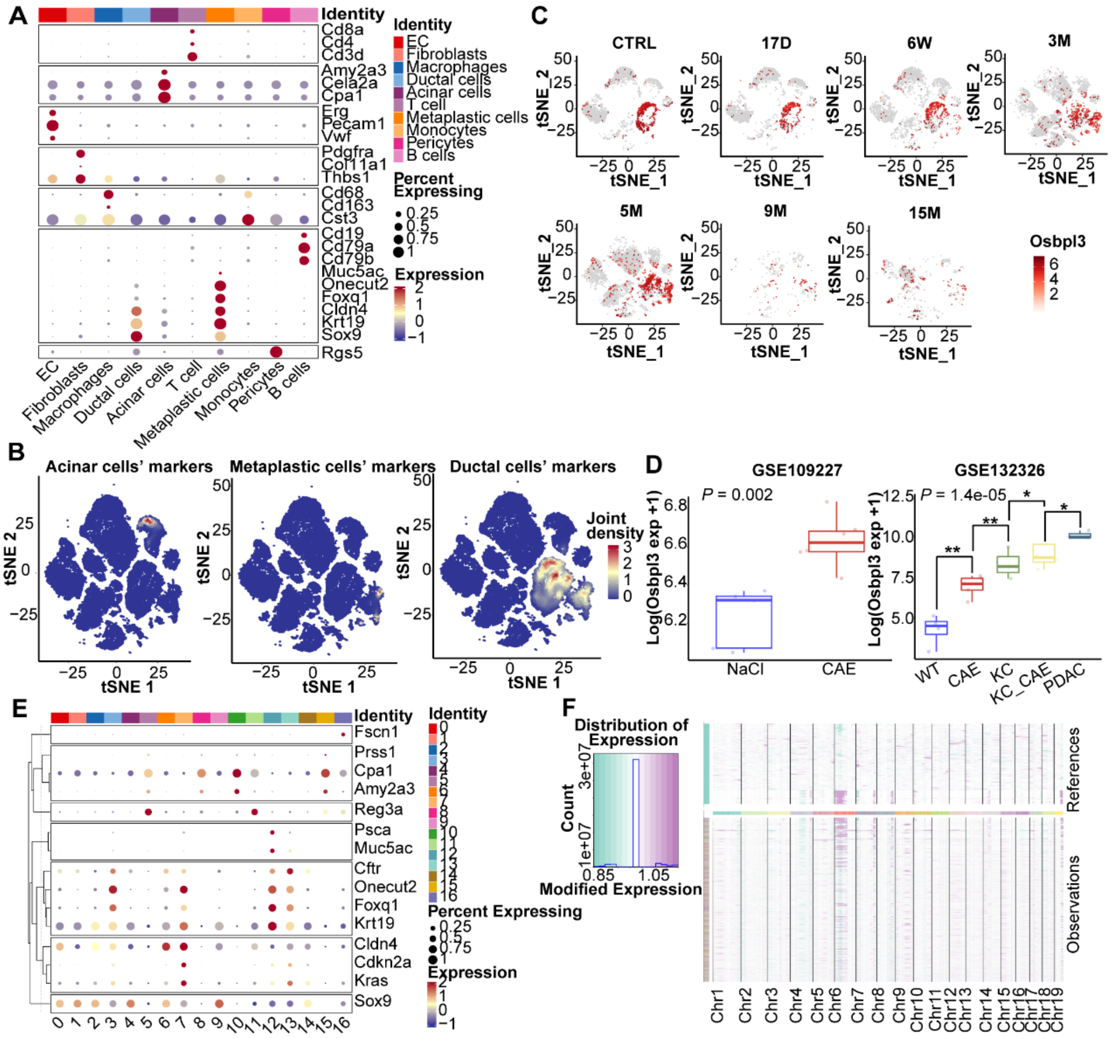
A
Identity
Cd8a
Cd4
Cd3d
Amy2a3
Cela2a
Cpa1
Erg
Pecam1
Vwf
Pdgfra
Col11a1
Thbs1
Cd68
Cd163
Cst3
Cd19
Cd79a
Cd79b
Muc5ac
Onecut2
Foxq1
Cldn4
Krt19
Sox9
Rgs5
EC
Fibroblasts
Macrophages
Ductal cells
Acinar cells
T cell
Metaplastic cells
Monocytes
Pericytes
B cells
Percent Expressing
0.25
0.5
0.75
1
Expression
2
1
0
−1
C
CTRL
17D
6W
3M
5M
9M
15M
tSNE_1
tSNE_2
Osbpl3
6
4
2
B
Acinar cells' markers
Metaplastic cells' markers
Ductal cells' markers
tSNE 1
tSNE 2
Joint density
D
GSE109227
GSE132326
P = 0.002
P = 1.4e-05
Log(Osbpl3 exp +1)
NaCl
CAE
WT
CAE
KC
KC_CAE
PDAC
E
Identity
Fscn1
Prss1
Cpa1
Amy2a3
Reg3a
Psca
Muc5ac
Cftr
Onecut2
Foxq1
Krt19
Cldn4
Cdkn2a
Kras
Sox9
Percent Expressing
Expression
F
Distribution of Expression
Count
Modified Expression
References
Observations
Chr1
Chr2
Chr3
Chr4
Chr5
Chr6
Chr7
Chr8
Chr9
Chr10
Chr11
Chr12
Chr13
Chr14
Chr15
Chr16
Chr17
Chr18
Chr19

Figure S2. Expression of Osbpl3 increases progressively during PDA initiation and progression.

(**A**) Dot plot showing the percentage of expressed cells and the average expression levels of canonical marker genes for major cell types across 10 distinct cell clusters (cohort: GSE141017).

(**B**) Density distribution of signature gene sets for acinar cells (Cpa1, Cela2a, and Amy2a), ductal cells (Krt19, Sox9, and Cldn4), and metaplastic cells (Onecut2, Foxq1, and Muc5ac) across different cell subpopulations

(**C**) Single-cell expression of Osbpl3 across different cell subpopulations at various time points from the GSE141017 dataset, which models PDA progression in the KC mouse model.

(**D**) OSBPL3 expression levels in normal, pancreatitis, and PDA samples from the GSE109227 and GSE132326 datasets.

(**E**) Bubble plot depicting the expression of marker genes for acinar, ductal, and malignant cells across different epithelial subpopulations.

(**F**) Copy number prediction of epithelial cells at various time points, with the CTRL group as a reference, based on the inferCNV algorithm.

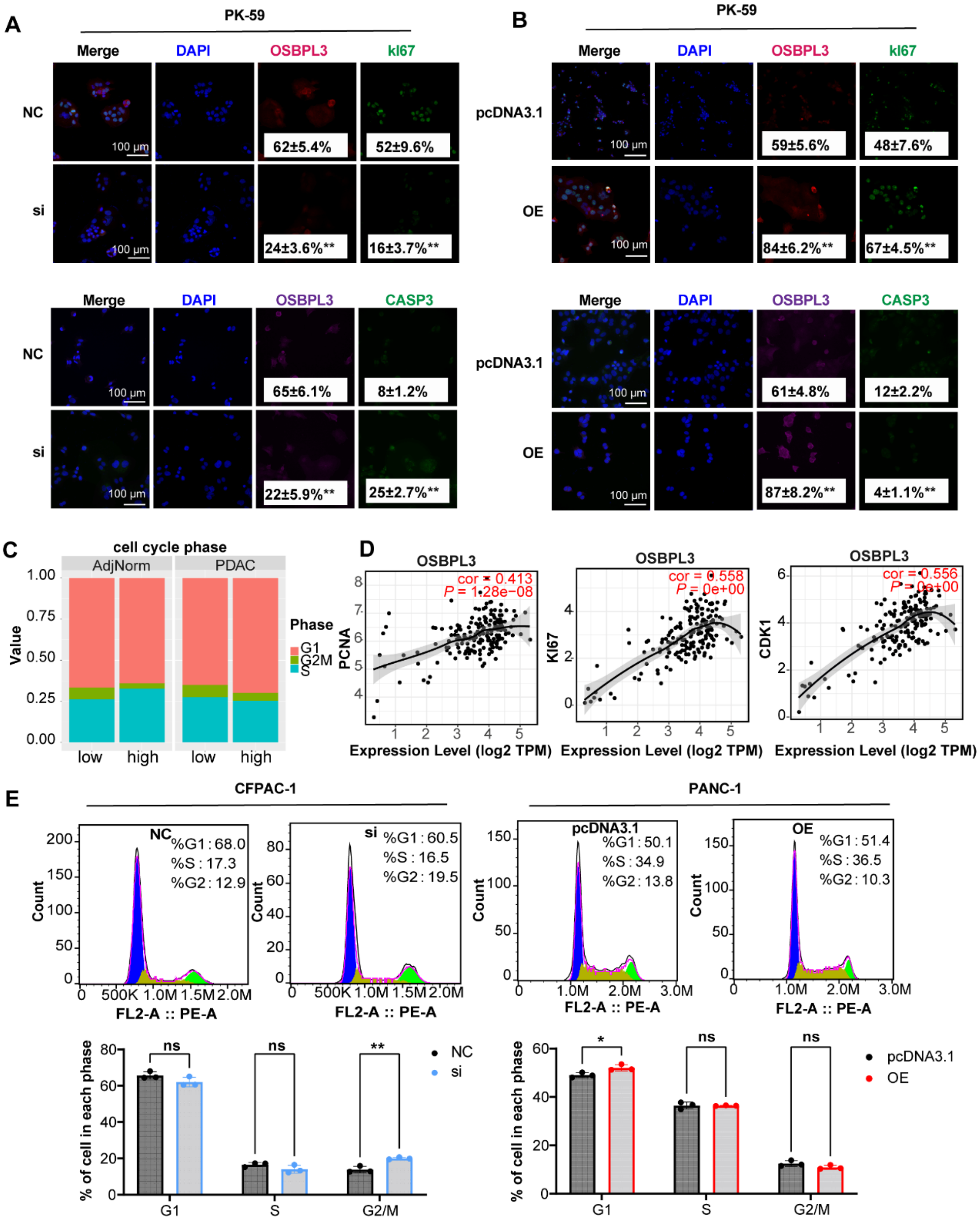
A
PK-59
Merge
DAPI
OSBPL3
kl67
NC
si
100 μm
62±5.4%
52±9.6%
24±3.6%**
16±3.7%**
Merge
DAPI
OSBPL3
CASP3
65±6.1%
8±1.2%
22±5.9%**
25±2.7%**
B
PK-59
pcDNA3.1
OE
59±5.6%
48±7.6%
84±6.2%**
67±4.5%**
61±4.8%
12±2.2%
87±8.2%**
4±1.1%**
C
cell cycle phase
AdjNorm
PDAC
Value
Phase
G1
G2M
S
low
high
D
OSBPL3
PCNA
cor = 0.413
P = 1.28e-08
KI67
cor = 0.558
P = 0e+00
CDK1
cor = 0.556
P = 0e+00
Expression Level (log2 TPM)
E
CFPAC-1
PANC-1
NC
%G1 : 68.0
%S : 17.3
%G2 : 12.9
si
%G1 : 60.5
%S : 16.5
%G2 : 19.5
pcDNA3.1
%G1 : 50.1
%S : 34.9
%G2 : 13.8
OE
%G1 : 51.4
%S : 36.5
%G2 : 10.3
Count
FL2-A :: PE-A
% of cell in each phase
ns
**
*
G1
S
G2/M

Figure S3. Role of OSBPL3 in PDA cell proliferation.

(**A**) and (**B**) Immunofluorescence staining in the PK-59 PDA cell line to assess the expression of proliferation (Ki67) and apoptosis (CASP3) markers before and after OSBPL3 knockdown or overexpression. Magnification: 20x. DAPI (blue) marks cell nuclei, red and purple fluorescence indicate OSBPL3 expression (mainly in the cytoplasm), and green fluorescence represents Ki67 (nuclear) and CASP3 (cytoplasmic) expression.

(**C**) Proportion of cells in different cell cycle phases in the human PDA and adjacent tissue single-cell dataset GSE155698. The stacked bar chart shows the percentage of cells at each cell cycle phase, grouped by high and low OSBPL3 expression levels, and further stratified by tumor or adjacent tissue origin.

(**D**) Correlation analysis between OSBPL3 expression and the proliferation-related genes PCNA, MKI67 and CDK1.

(**E**) Flow cytometry analysis of cell cycle changes following OSBPL3 knockdown or overexpression. The bar chart shows the differences in the proportions of cells at various cell cycle phases between the two groups.

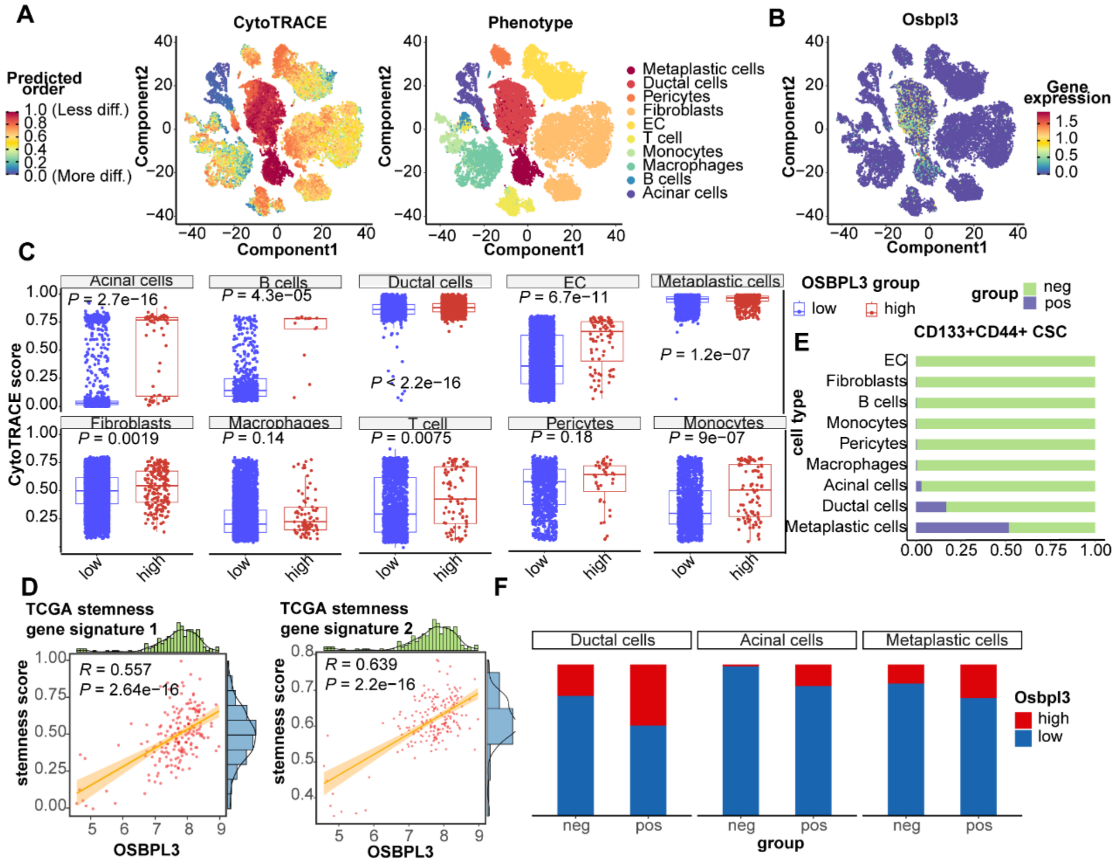
A
CytoTRACE
Phenotype
Predicted order
1.0 (Less diff.)
0.8
0.6
0.4
0.2
0.0 (More diff.)
Component2
Component1
Metaplastic cells
Ductal cells
Pericytes
Fibroblasts
EC
T cell
Monocytes
Macrophages
B cells
Acinar cells
B
Osbpl3
Gene expression
C
CytoTRACE score
Acinal cells P = 2.7e−16
B cells P = 4.3e−05
Ductal cells P < 2.2e−16
EC P = 6.7e−11
Metaplastic cells P = 1.2e−07
Fibroblasts P = 0.0019
Macrophages P = 0.14
T cell P = 0.0075
Pericytes P = 0.18
Monocytes P = 9e−07
low
high
OSBPL3 group
group neg pos
E
CD133+CD44+ CSC
cell type
D
TCGA stemness gene signature 1
R = 0.557
P = 2.64e−16
TCGA stemness gene signature 2
R = 0.639
P = 2.2e−16
stemness score
OSBPL3
F
Ductal cells
Acinal cells
Metaplastic cells
Osbpl3
high
low
neg
pos
group

Figure S4. The role of OSBPL3 in tumor stemness in PDA.

(**A**) Dimensionality reduction plot based on CytoTRACE pseudotime analysis showing stemness prediction scores for single-cell samples from the KC pancreatic mouse model.

(**B**) The plot represents CytoTRACE predicted scores, along with OSBPL3 gene expression.

(**C**) Faceted boxplot showing the CytoTRACE predicted stemness scores for cells with high or low OSBPL3 expression across different cell subpopulations.

(**D**) Correlation between the GSVA scores of two tumor stemness gene sets and OSBPL3 expression in TCGA-PAAD pancreatic cancer transcriptomic data.

(**E**) and (**F**) Bar chart displaying the proportion of tumor stem cells based on CD133 and CD44 marker gene annotations in various cell subpopulations, as well as the proportion of tumor stem cells in acinar cells, ductal cells, and metaplastic cells, comparing OSBPL3 high and low expression groups.

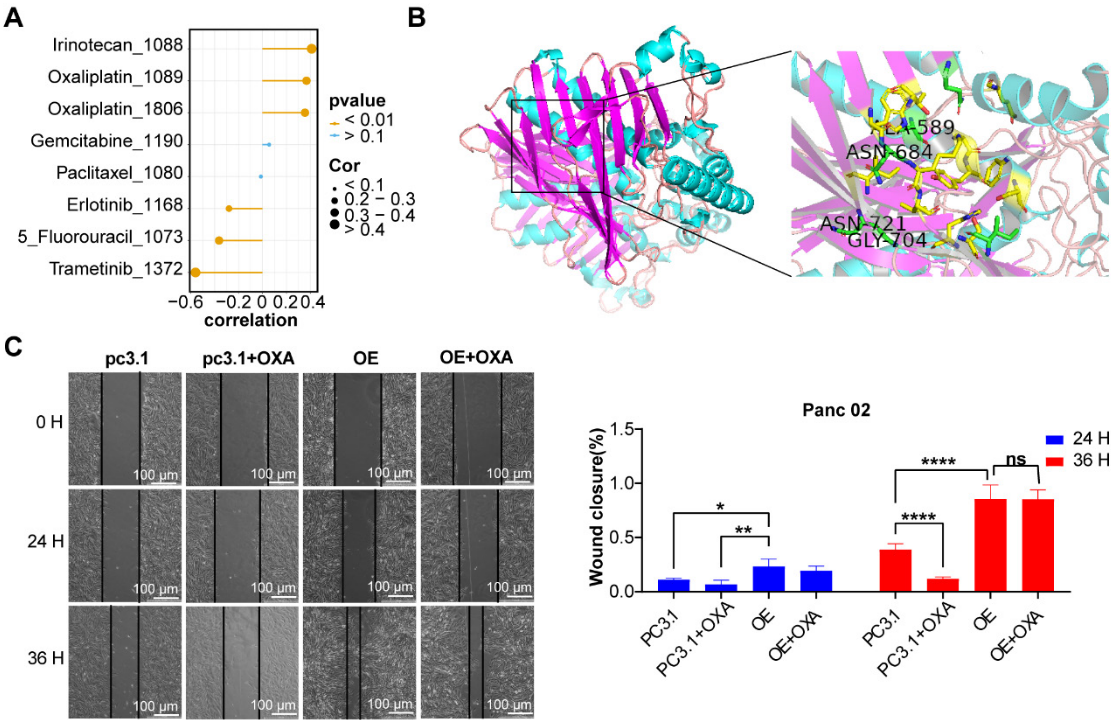

Figure S5. High expression of OSBPL3 reverses the inhibitory effect of oxaliplatin on PDA cell migration.

(**A**) Lollipop plot showing the correlation between OSBPL3 expression and the IC50 values of common pancreatic cancer chemotherapy drugs. Orange represents *P*-value < 0.05, while blue represents *P*-value > 0.1. The length of the line and the size of the circle are positively correlated with the correlation value.

(**B**) Molecular docking prediction of the binding sites between OSBPL3 and oxaliplatin.

(**C**) Representative images from the wound healing assay to assess cell migration in the Panc02 PDA cell line, following changes in Osbpl3 expression and oxaliplatin treatment. The bar chart shows statistical analysis of cell migration rates at 12 and 24 h time points. Blue and red represent migration rates at 12 and 24 h, respectively. ANOVA and multiple t-tests were performed. ns indicates no significant difference, **P* < 0.05, ***P* < 0.01, ****P* < 0.001, *****P* < 0.0001.

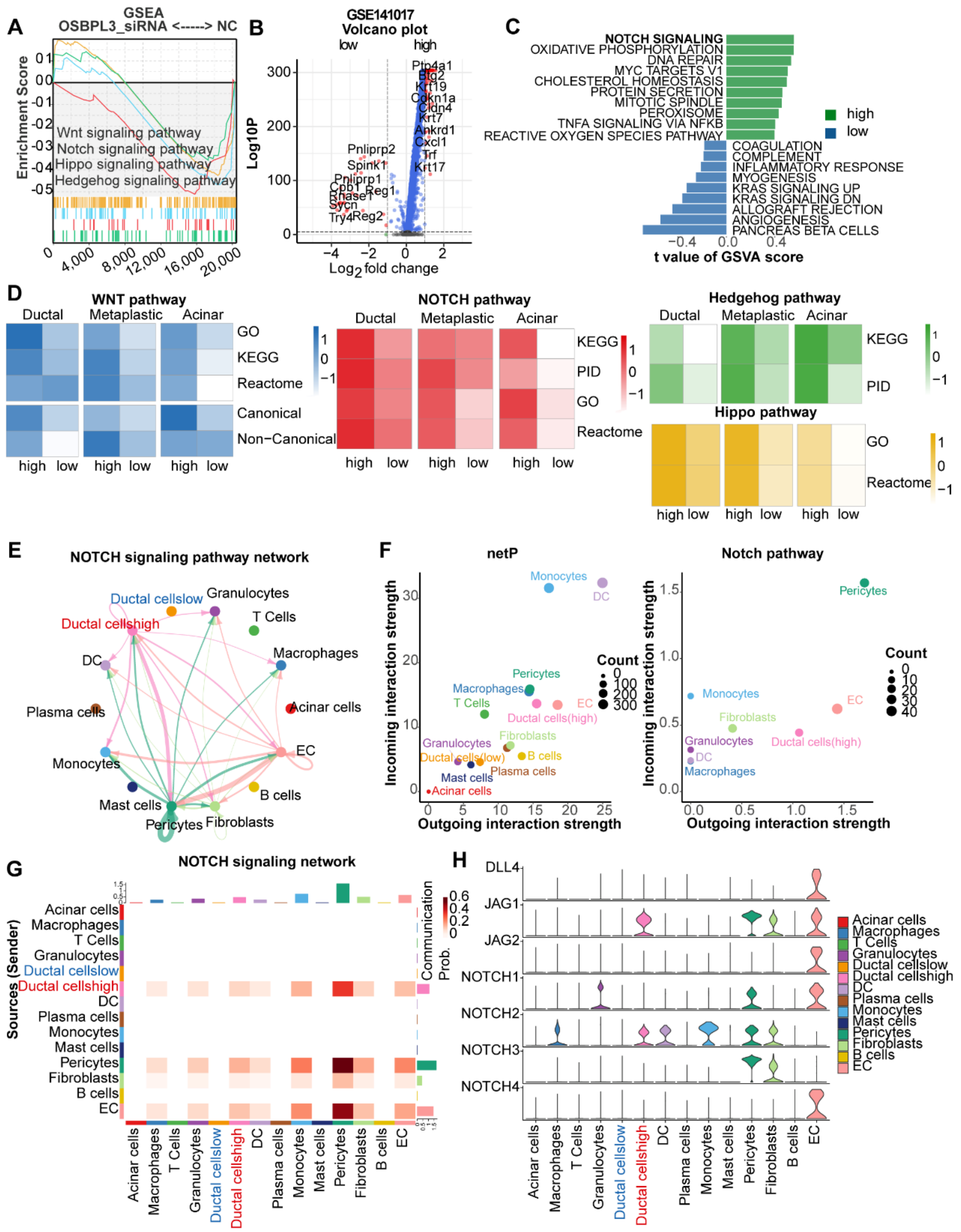
A
GSEA
OSBPL3_siRNA <-----> NC
Enrichment Score
Wnt signaling pathway
Notch signaling pathway
Hippo signaling pathway
Hedgehog signaling pathway
B
GSE141017
Volcano plot
low
high
Log10P
Log2 fold change
C
NOTCH SIGNALING
OXIDATIVE PHOSPHORYLATION
DNA REPAIR
MYC TARGETS V1
CHOLESTEROL HOMEOSTASIS
PROTEIN SECRETION
MITOTIC SPINDLE
PEROXISOME
TNFA SIGNALING VIA NFKB
REACTIVE OXYGEN SPECIES PATHWAY
COAGULATION
COMPLEMENT
INFLAMMATORY RESPONSE
MYOGENESIS
KRAS SIGNALING UP
KRAS SIGNALING DN
ALLOGRAFT REJECTION
ANGIOGENESIS
PANCREAS BETA CELLS
t value of GSVA score
D
WNT pathway
NOTCH pathway
Hedgehog pathway
Hippo pathway
Ductal
Metaplastic
Acinar
GO
KEGG
Reactome
Canonical
Non−Canonical
PID
high
low
E
NOTCH signaling pathway network
F
netP
Notch pathway
Incoming interaction strength
Outgoing interaction strength
Count
G
NOTCH signaling network
Sources (Sender)
Communication Prob.
H
DLL4
JAG1
JAG2
NOTCH1
NOTCH2
NOTCH3
NOTCH4
Acinar cells
Macrophages
T Cells
Granulocytes
Ductal cellslow
Ductal cellshigh
DC
Plasma cells
Monocytes
Mast cells
Pericytes
Fibroblasts
B cells
EC

Figure S6. OSBPL3 overexpression group shows significant activation of pathways such as NOTCH

(**A**) GSEA analysis of stemness-related regulatory pathways in CFPAC-1 cells, comparing to NC and OSBPL3 Si groups.

(**B**) Volcano plot illustrating differentially expressed genes between the high and low OSBPL3 expression groups in epithelial cell subpopulations extracted from the KC mouse single-cell dataset (GSE141017).

(**C**) Waterfall plot presenting the enrichment analysis of HALLMARK pathways for the high and low OSBPL3 expression groups.

(**D**) GSVA scores for stemness-regulating pathways such as NOTCH and WNT in different cell subpopulations based on high or low OSBPL3 expression.

(**E**) Cell-cell communication network analysis of NOTCH pathway-related interactions between different cell types in PDA tissues.

(**F**) Cell communication intensity for different cell types, along with the communication strength related to the NOTCH pathway.

(**G**) Heatmap displaying NOTCH pathway-related communication networks when different cell types act as signal senders or receivers.

(**H**) Violin plot illustrating the distribution of gene expression for NOTCH signaling-related genes across different cell types.

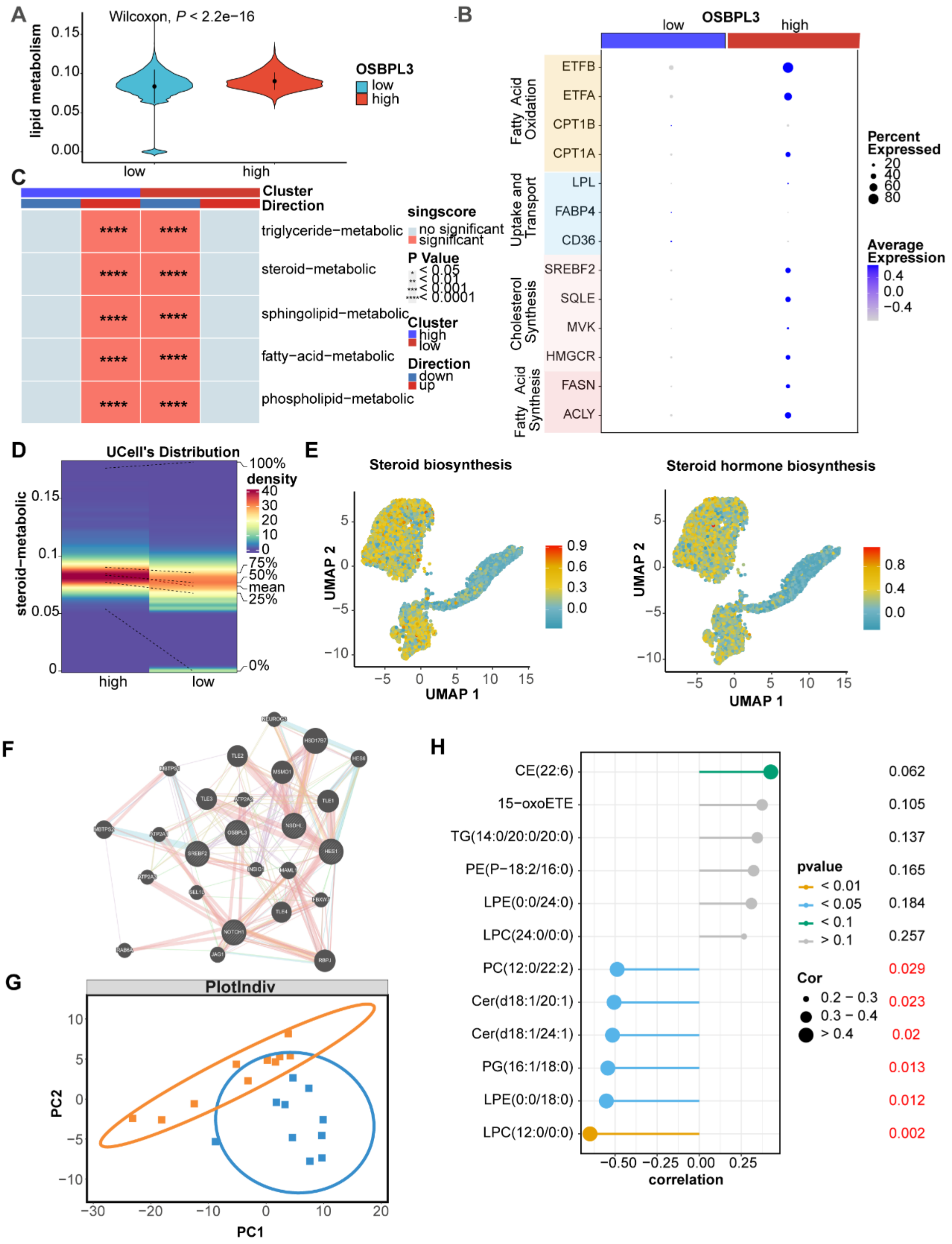
A
Wilcoxon, P < 2.2e−16
lipid metabolism
0.15
0.10
0.05
0.00
low
high
OSBPL3
low
high
B
OSBPL3
low
high
Fatty Acid Oxidation
ETFB
ETFA
CPT1B
CPT1A
Uptake and Transport
LPL
FABP4
CD36
Cholesterol Synthesis
SREBF2
SQLE
MVK
HMGCR
Fatty Acid Synthesis
FASN
ACLY
Percent Expressed
20
40
60
80
Average Expression
0.4
0.0
−0.4
C
Cluster
Direction
triglyceride−metabolic
steroid−metabolic
sphingolipid−metabolic
fatty−acid−metabolic
phospholipid−metabolic
singscore
no significant
significant
P Value
< 0.05
< 0.01
< 0.001
< 0.0001
Cluster
high
low
Direction
down
up
D
UCell's Distribution
steroid−metabolic
100%
density
75%
50%
mean
25%
0%
high
low
E
Steroid biosynthesis
Steroid hormone biosynthesis
UMAP 1
UMAP 2
F
G
PlotIndiv
PC1
PC2
H
CE(22:6)
15−oxoETE
TG(14:0/20:0/20:0)
PE(P−18:2/16:0)
LPE(0:0/24:0)
LPC(24:0/0:0)
PC(12:0/22:2)
Cer(d18:1/20:1)
Cer(d18:1/24:1)
PG(16:1/18:0)
LPE(0:0/18:0)
LPC(12:0/0:0)
0.062
0.105
0.137
0.165
0.184
0.257
0.029
0.023
0.02
0.013
0.012
0.002
pvalue
< 0.01
< 0.05
< 0.1
> 0.1
Cor
0.2 − 0.3
0.3 − 0.4
> 0.4
correlation

Figure S7. OSBPL3 Overexpression Contributes to Increased Cholesterol synthesis in PDA

(**A**) Violin plot showing lipid metabolism scores between OSBPL3 high and low expression groups in the single-cell dataset GSE155698. Lipid metabolism scores were calculated using the AddModuleScore algorithm. Statistical significance was assessed using the two-sided Wilcoxon rank-sum test. $^{*}P < 0.05$, $^{**}P < 0.01$, $^{***}P < 0.001$, $^{****}P < 0.0001$.

(**B**) Heatmap showing temporal regulation of lipid metabolism gene sets—including steroid metabolism, fatty acid metabolism, and phospholipid metabolism—based on irGSEA scoring across different time points. Gene set enrichment significance was determined by permutation-adjusted *p*-values (FDR < 0.25 considered significant).

(**C**) Dot plot showing expression of representative genes in cholesterol and fatty acid synthesis pathways stratified by OSBPL3 expression level. Dot size indicates the proportion of expressing cells; dot color reflects average scaled expression.

(**D**) Density plot showing the distribution of steroid biosynthesis gene expression in the high vs. low OSBPL3 expression groups.

(**E**) UMAP dimensionality reduction plots for steroid synthesis, and steroid hormone synthesis pathways.

(**F**) Protein-protein interaction network for OSBPL3 analyzed using the GeneMANIA website.

(**G**) PCA plot showing the spatial distribution of two groups of samples after PLS-DA analysis.

(**H**) Lolipop Plot displaying the top 20 metabolites most strongly correlated with OSBPL3 across different samples.

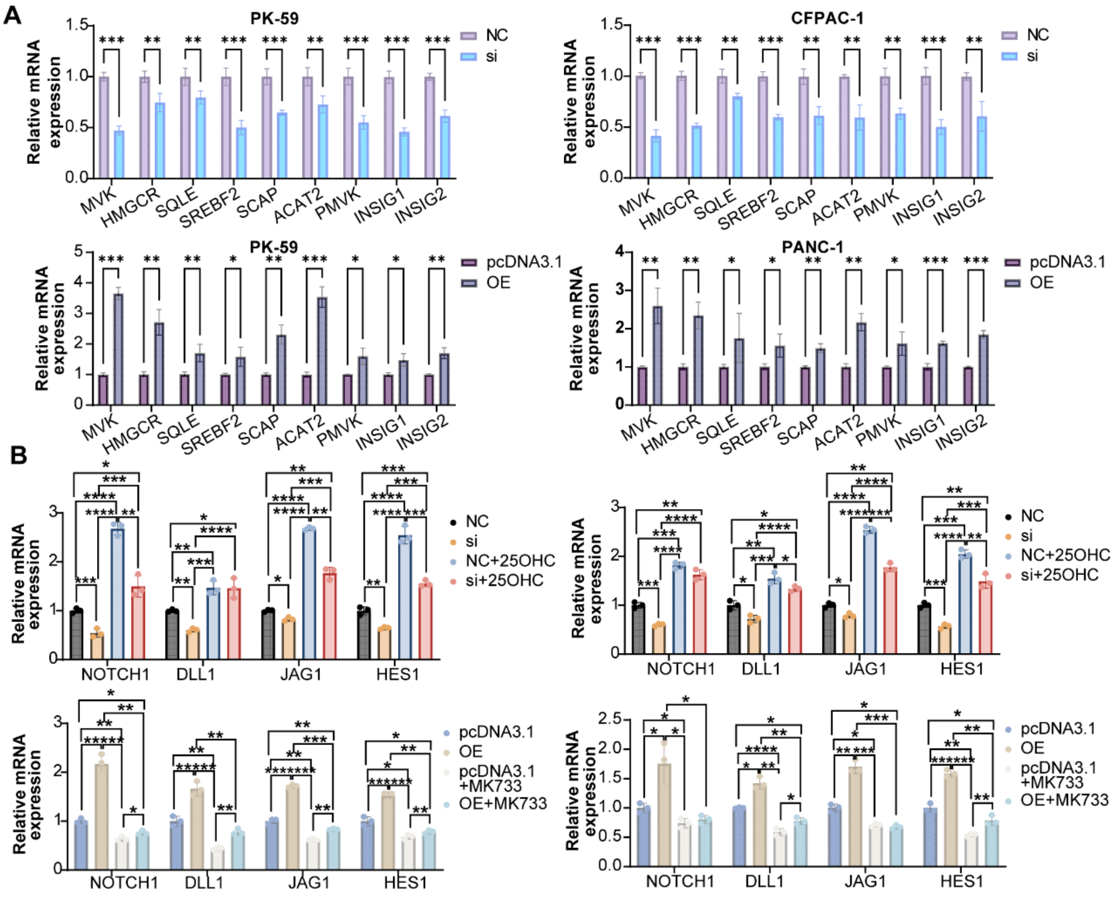

Figure S8. OSBPL3 regulates cholesterol metabolism and NOTCH signaling pathway activity.

(**A**) qRT-PCR analyses were performed to assess the expression levels of key cholesterol related genes in PDA cells before and after OSBPL3 knockdown or overexpression (n = 3).

(**B**) Quantitative real-time PCR (qPCR) analysis of NOTCH pathway related genes NOTCH1, JAG1 and HES1 in OSBPL3-knockdown (CFPAC-1 and PK-59) with 25-hydroxycholesterol (25-OHC, 2.5 μM, 24 h) and OSBPL3-overexpressing (PANC-1 and PK-59) with simvastatin (15 nM, 24 h) treated pancreatic cancer cells. β-actin was used as an internal control.

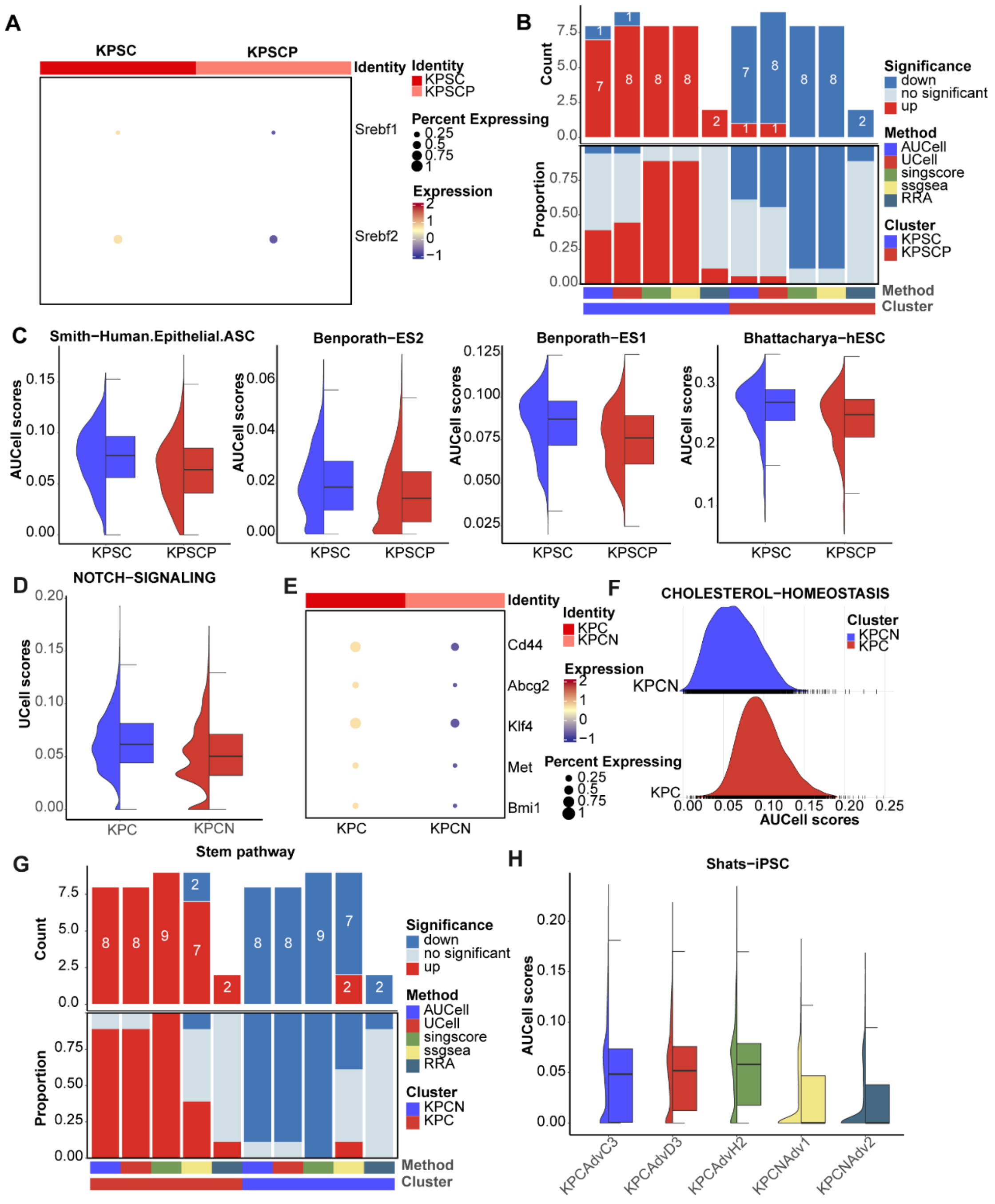
A
KPSC
KPSCP
Identity
Srebf1
Srebf2
Percent Expressing
0.25
0.5
0.75
1
Expression
B
Significance
down
no significant
up
Method
AUCell
UCell
singscore
ssgsea
RRA
Cluster
Count
Proportion
C
Smith−Human.Epithelial.ASC
Benporath−ES2
Benporath−ES1
Bhattacharya−hESC
AUCell scores
D
NOTCH−SIGNALING
UCell scores
KPC
KPCN
E
Cd44
Abcg2
Klf4
Met
Bmi1
F
CHOLESTEROL−HOMEOSTASIS
AUCell scores
G
Stem pathway
H
Shats−iPSC
KPCAdvC3
KPCAdvD3
KPCAdvH2
KPCNAdv1
KPCNAdv2

Figure S9. Downregulation of key genes in cholesterol synthesis Inhibits the NOTCH pathway and tumor stemness

(**A**) Bubble plot showing the expression levels of OSBPL3 in KPSC (before) and KPSCP (after) samples from KPC mice following treatment with Perhexiline maleate to inhibit Srebf2.

(**B**) Bar chart displaying the scores for four stemness-related datasets in the KPSC and KPSCP groups, derived from five different algorithms. Red indicates upregulated gene sets, and blue indicates downregulated gene sets.

(**C**) Half violin plot illustrating the AUCell scores for four stemness-related datasets across different samples.

(**D**) Half violin plot showing the UCell scores for the NOTCH pathway in the KPC and KPCN groups before (KPC) and after (KPCN) conditional inactivation of the cholesterol synthesis key enzyme, Nsdhl.

(**E**) Bubble plot presenting the expression levels of stemness-related genes in the KPCN group (KPCNAdv1 and KPCNAdv2) and control KPC group (KPCAdvD3, KPCAdvC3, and KPCAdvH2) samples, following conditional inactivation of Nsdhl.

(**F**) Density plot showing the distribution density of AUCell scores for sterol metabolism-related pathways in KPC and KPCN groups.

(**G**) Bar chart displaying the scores for nine stemness-related datasets in the KPC and KPCN groups, based on five different algorithms. Red represents upregulated gene sets, while blue represents downregulated gene sets.

(**H**) Half violin plot illustrating the AUCell scores for three stemness-related datasets across different samples.
